# A Series of Solution-Stable Heterometallic Molecular Phosphonates with {Co_9_Dy}, {Co_7_Dy}, and {Co_6_Dy} Cores

**DOI:** 10.1021/acs.inorgchem.6c01194

**Published:** 2026-05-08

**Authors:** Iaroslav Doroshenko, Tomas Pokorny, Lucie Simonikova, Zdenek Moravec, Jaromír Marek, Ján Vančo, Zdeněk Trávníček, Jiri Pinkas

**Affiliations:** † Department of Chemistry, Faculty of Science, 37748 Masaryk University, Kotlarska 2, Brno CZ-61137, Czech Republic; ‡ Regional Center of Advanced Technologies and Materials, Czech Advanced Technology and Research Institute, 48207 Palacký University, Šlechtitelů 27, Olomouc CZ-779 00, Czech Republic

## Abstract

A family of three heterometallic molecular phosphonate clusters with {Co_*x*_Dy} (*x* = 6, 7, 9) cores was synthesized using the Schiff-base-derived phosphonate ligand HSAA^2–^. The reactions of CoCl_2_·6H_2_O and DyCl_3_·6H_2_O with HSAA^2–^ under various conditions afforded the polynuclear complexes [Co_9_Dy­(SAA)_6_Cl_3_] (**1**), [Na_2_Co_7_Dy­(SAA)_6_(SA)] (**2**), and [Na_3_Co_6_Dy­(SAA)_6_] (**3**), which are based on a common {Co_6_Dy} phosphonate core further stabilized by additional cobalt or sodium cations. The complexes were found to be thermally stable up to 250 °C, soluble, and stable in polar aprotic solvents. Direct current (dc) magnetic susceptibility measurements recorded over the temperature range 2–300 K were interpreted using an effective-spin model. The analysis revealed an isotropic Co^2+^ contribution and a strong axial Dy^3+^ response consistent with Ising-type magnetic behavior. Despite the presence of strongly anisotropic Dy^3+^ centers, alternating current (ac) magnetic measurements revealed negligible slow relaxation of magnetization, even under an external magnetic field.

## Graphical abstract

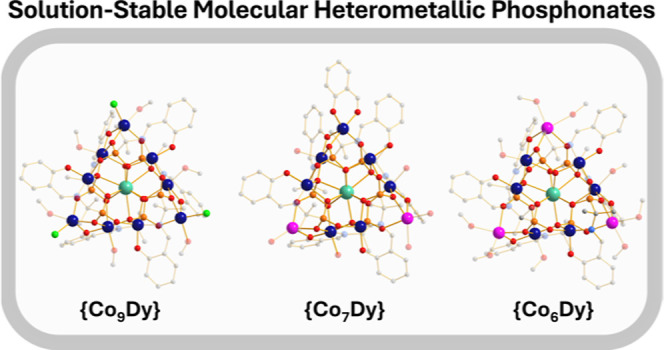

## Introduction

Molecular heterometallic 3d–4f complexes remain at the forefront of the field of molecular magnetism because they combine different spin carriers and multiple exchange pathways available through the metal centers with spin–orbit coupling and the large single-ion anisotropy of lanthanides. Their different chemistries and stereochemical requirements, combined with the ferromagnetic arrangement of anisotropic metal centers, resulted in synergy that has delivered some of the most compelling routes toward molecules with slow magnetic relaxation (SMMs) and related cooperative phenomena.^1–18^ The major challenge lies in elucidating exchange and dipolar interactions between 3d and 4f ions, as demonstrated by relatively simple structural motifs featuring {Co_2_Ln_2_} butterfly cores.^19^

Phosphonate ligands are particularly attractive building blocks for robust multinuclear architectures because they provide strong, polyvalent P–O donor motifs with multiple bridging modes, high thermal/chemical stability, and the ability to enforce unusual topologies not easily accessible with other ligands, such as carboxylates or β-diketonates.^20–22^ Nevertheless, the very same strong coordination ability that makes phosphonates robust often results in insoluble polymeric or extended phases, rendering the isolation of discrete molecular complexes a synthetic challenge that can be overcome in several ways.^21^ This limitation has slowed the systematic exploration of phosphonate-based complexes in the field of molecular magnetism, in contrast to other ligand systems, such as carboxylates.^22^ The first 3d-4f phosphonate cluster displaying slow relaxation of the magnetization and SMM behavior with a small energy barrier contained a {Cu_24_Dy_8_} core.^1^

Our Schiff-base-modified aminophosphonate ligand HSAA^2–^ (Chart 1) was found to successfully overcome this issue. The combination of a phosphonate group with auxiliary O/N donor sites promotes intramolecular chelation and controlled bridging, thereby favoring the formation of well-defined molecular clusters rather than extended networks.^23–25^ In this regard, HSAA^2–^ represents a rare ligand platform that allows the targeted assembly of multinuclear molecular phosphonates under mild synthetic conditions.

**Chart 1:**
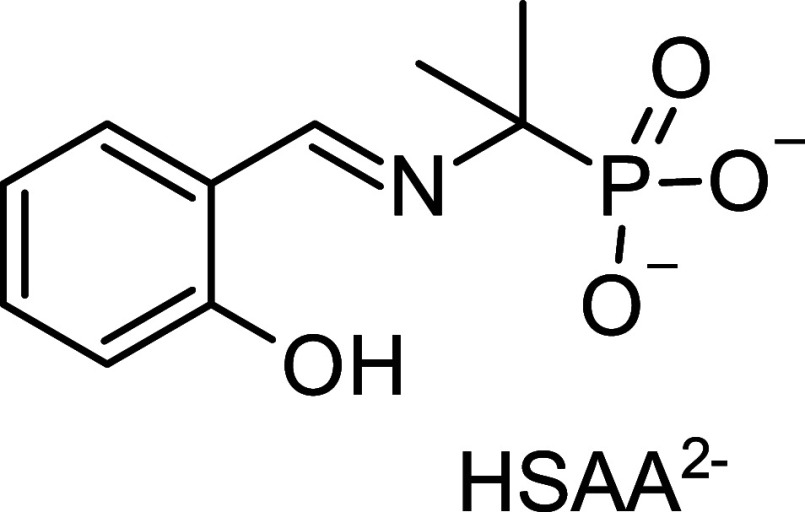
Schematic Representation of the HSAA^2–^ Ligand Used in This Study

Another persistent difficulty in 3d–4f cluster chemistry is the selective formation of true heterometallic species. Simple mixing of transition-metal and lanthanide salts with bridging ligands typically yields either separate homometallic complexes or poorly defined mixtures. The controlled creation of a single, structurally robust heterometallic molecular cluster is therefore nontrivial and represents an important achievement.^18,26,27^ In our case, the use of the HSAA^2–^ ligand (Chart 1) has already demonstrated effectiveness in forming {Co_2_Gd_4_} mixed-metal complexes.^25^ The present study shows that employing the HSAA^2–^ ligand enables the selective assembly of Co–Dy clusters with different nuclearities ({Co_9_Dy}, {Co_7_Dy}, and {Co_6_Dy}), each featuring a conserved {Co_6_Dy} phosphonate core and displaying the ability of the ligand to enforce heterometallic integration within a single complex molecule.

Co–Dy assemblies are especially appealing because high-spin Co^2+^ centers supply both exchange interactions and substantial orbital contributions, while Dy^3+^ (^6^H_15/2_) provides a typically large axial anisotropy that can produce pronounced slow relaxation when the local crystal field is favorable.^28^ Notable examples illustrating the potential of Co–Dy heterometallic systems include high-nuclearity wheels and other heterometallic motifs that show SMM behavior under appropriate structural/electronic conditions.^29–31^ The chemistry of 3d–4f metal phosphonates remains relatively underexplored, with only a limited number of reported examples in the literature.^25,32–36^ Achieving predictable magnetic outcomes in 3d–4f phosphonate chemistry remains challenging: small changes in nuclearity, peripheral counterions, or bridging topology can significantly impact the resulting magnetic properties.

Multinuclear homo- or heterometallic complexes that are soluble and stable in solutions have potential for catalytic applications.^37,38^ The presence of robust polynuclear cores, labile peripheral ligands (Cl^–^, MeOH, H_2_O), and solution stability make these clusters attractive as precursors for materials, including various heterogeneous catalytic systems.^39–42^ Although catalytic studies were beyond the scope of the present work, the demonstrated solution stability of the {Co_*x*_Dy} cores highlights their potential to serve as versatile platforms not only for molecular magnetism but also for catalytic transformations and as precursors for materials.

In this study, we focused on assembling multinuclear Co–Dy phosphonate clusters using the HSAA^2–^ ligand (Chart 1), which combines a strong phosphonate donor group with additional O/N coordination sites. By varying the synthetic conditions, we isolated three new neutral complexes: [Co_9_Dy­(SAA)_6_Cl_3_] (**1**), [Na_2_Co_7_Dy­(SAA)_6_(SA)] (**2**), and [Na_3_Co_6_Dy­(SAA)_6_] (**3**). All three compounds feature a central {Co_6_Dy} core but differ in the peripheral charge-balancing ions, resulting in closely related {Co_9_Dy}, {Co_7_Dy}, and {Co_6_Dy} motifs. Their comprehensive characterization was performed by using single-crystal X-ray diffraction, IR, ESI-MS, X-ray photoelectron spectroscopy (XPS), thermogravimetric analysis, and magnetometry. Our results demonstrate the structural versatility of this ligand system and provide insights into the delicate balance between exchange interactions and single-ion anisotropy in Co–Dy assemblies. Moreover, the observed solution stability and modular structural motifs suggest that these clusters can be employed in homogeneous catalysis and as molecular precursors for extended networks.

## Results and Discussion

Reactions of cobalt and dysprosium chlorides hydrate with the HSAA^2–^ ligand (**1**, **2**, and **3**), salicylaldehyde (SA, **2**), and sodium hydroxide in slightly different stoichiometric ratios in methanol solution led to the formation of three new complexes: [Co_9_Dy­(SAA)_6_Cl_3_] (**1**), [Na_2_Co_7_Dy­(SAA)_6_(SA)] (**2**), and [Na_3_Co_6_Dy­(SAA)_6_] (**3**). Complexes **1** and **2** were also prepared by reacting the molecular heptanuclear cobalt phosphonate complex [Co_7_(SAA)_2_(HSAA)_4_], previously described,^23^ with DyCl_3_ and NaOH in acetone and methanol solutions (Method B), respectively. The synthesis of complex **1** also demonstrated the feasibility of preparing the complex using a ligand formed in situ (Method C, Supporting Information), which represents an advantageous approach for scaling up the synthesis.

The structures of complexes **1**–**3** were determined by single-crystal X-ray diffraction analysis of suitable crystals. The complexes crystallized in the monoclinic space groups *P*2_1_/*n* and *P*2_1_/*c* for **1** and **2**, respectively, and the triclinic space group *P* 1̅ (**3**). Crystallographic data and structure refinement parameters are listed in Table 1.

**Table 1:** Selected Crystallographic Data and Structure Refinement Parameters for **1**, **2**, and **3** ^a^

parameters	**1**	**2**	**3**
formula^a^	C_65.61_H_82.84_Cl_3_Co_9_DyN_6_O_29.61_P_6_	C_67_H_71_Co_7_DyN_6_Na_2_O_32_P_6_	C_69_H_93_Co_6_DyN_6_Na_3_O_33_P_6_
fw (g mol^–1^)	2414.41	2279.10	2305.36
cryst syst	monoclinic	monoclinic	triclinic
space group	*P*2_1_/*n*	*P*2_1_/*c*	*P* 1̅
*a* (Å)	18.3054(3)	15.8439(2)	16.74683(13)
*b* (Å)	21.9150(3)	28.1811(6)	17.86874(17)
*c* (Å)	26.4613(4)	22.9076(4)	18.84983(14)
α (deg)	90	90	85.5061(7)
β (deg)	109.526(2)	100.8279(16)	83.6659(6)
γ (deg)	90	90	87.8184(7)
*V* (Å^3^)	10004.8(3)	10046.1(3)	5586.58(8)
*Z*	4	4	2
*T* (K)	120.01(10)	120(2)	293(2)
δ_calc_ (g cm^–3^)	1.603	1.507	1.370
*F*(000)	4822	4552	2328
μ(Mo–K_α_) (mm^–1^)	2.434	2.036	1.694
θ range of data collection (deg)	1.858–25.000	1.871–24.999	1.854–31.759
meas. refl.	77791	81704	76551
unique refl. (*R*_int_)	17597 (0.0460)	17652 (0.0472)	31763 (0.0198)
no. of param	1130	1102	1148
GOF on *F* ^2^ ^b^	1.087	1.138	1.008
*R*_1_ [*I* > 2σ(*I*)]	0.0388	0.0846	0.0333
w*R*_2_ (all data)^c^	0.1034	0.1949	0.0919
Δρ_max_ (e Å^–3^)	1.684	3.387	1.837
Δρ_min_ (e Å^–3^)	–0.933	–1.975	–1.730
CCDC	2482594	2482595	2482596

a The molecular formulas and molecular weights corresponding to the data obtained from the single-crystal X-ray analysis are used in this table. These formulas could differ from the ones obtained by elemental analysis of dried samples.

b $GOF={\left(\frac{\sum \left[w{\left(F_{o}^{2}-F_{c}^{2}\right)}^{2}\right]}{\left(N_{o}-N_{p}\right)}\right)}^{1/2}$ .

c $R_{1}=\frac{\sum \left|\left|F_{o}\right|-\left|F_{c}\right|\right|}{\sum \left|F_{o}\right|}$ ; $wR_{2}={\left(\frac{\sum \left[w{\left(F_{o}^{2}-F_{c}^{2}\right)}^{2}\right]}{\sum \left[w{\left(F_{o}^{2}\right)}^{2}\right]}\right)}^{1/2}$ with *w* ^–1^ = σ^2^(*F*_o_^2^)+(*a*P)^2^+bP; $P=\frac{2F_{c}^{2}+\max F_{o}^{2}}{3}$ .

The three obtained complexes **1–3** are structurally related and belong to the same structural family. All of these feature a central {Co_6_Dy} metallophosphonate core. This core is further completed by the additional three cobalt cations in complex **1**, two sodium and one cobalt cations in complex **2**, and three sodium cations in complex **3**. This leads to the formation of {Co_9_Dy}, {Co_7_Dy}, and {Co_6_Dy} 3d–4f cores in complexes **1**, **2**, and **3**, respectively. The molecular structures of complexes **1**–**3** are shown in Figure 1. The central {Co_6_Dy} core is formed solely through the coordination of six SAA^3–^ phosphonate ligands to the metal centers (Figures 1 and 2).

**Figure 1:**
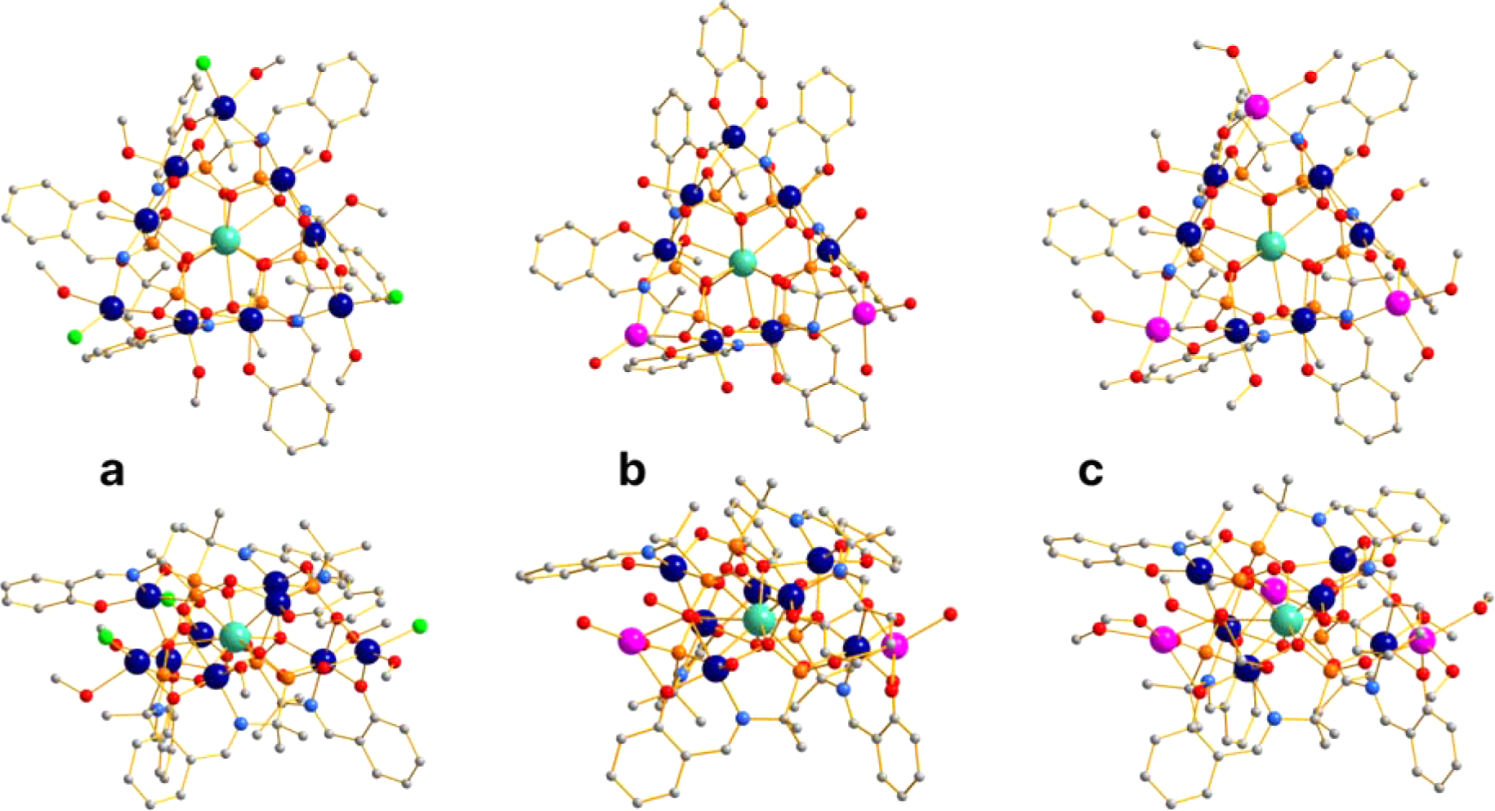
Ball-and-stick representation of the structural models of **1** (a), **2** (b), and **3** (c) in axial (top) and side (bottom) projections. Color code: Dy, turquoise; Co, blue; Na, magenta; P, orange; O, red; N, pale-blue; C, gray; and Cl, green. Hydrogen atoms were omitted for clarity.

**Figure 2:**
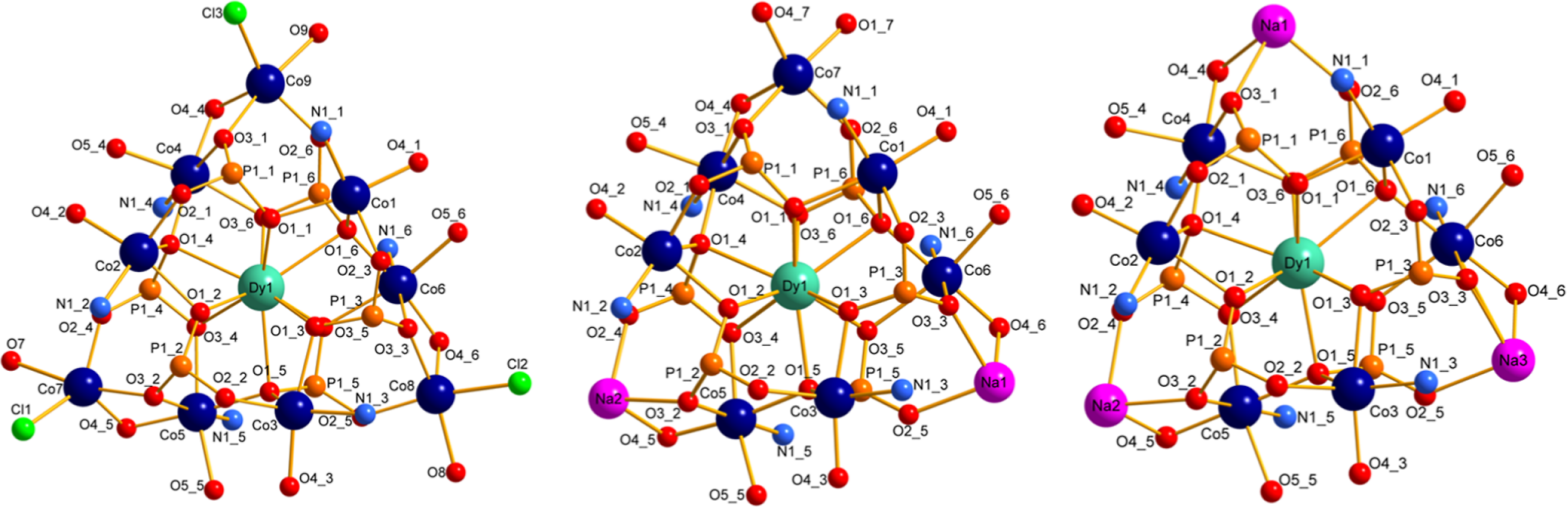
Ball-and-stick representation of the metallophosphonate cores of **1**, **2**, and **3** (from left to right). Bridging oxyphenyl oxygen atoms O_4_ are also depicted in the cores. Color code: Dy, turquois; Co, blue; Na, magenta; P, orange; and O red.

According to the bond valence sum (BVS),^43^ all cobalt ions are in 2+ and dysprosium ions are in 3+ oxidation states (Table S1). This is in good agreement with the Co XPS data (see below). As a result, the central {Co_6_Dy­(SAA)_6_} metallophosphonate core possesses a 3– charge. In complex **1**, three Co^2+^ cations coordinated by three chloride anions coordinate to the {Co_6_Dy­(SAA)_6_} core, introducing a 3+ charge, yielding a neutral molecule [Co_9_Dy­(SAA)_6_Cl_3_] (Figure 1a). In complex **2**, this 3– charge is balanced by two Na^+^ and one Co^2+^ cations. An additional 1+ charge is neutralized by the coordination of a 2-formylphenolate anion, leading to the formation of the neutral complex [Na_2_Co_7_Dy­(SAA)_6_(SA)] (Figure 1b). In complex **3**, the 3– charge of the core is compensated by three sodium cations, resulting in the neutral molecule [Na_3_Co_6_Dy­(SAA)_6_] (Figure 1c).

In all complexes **1**–**3**, the SAA^3–^ phosphonate ligands could be divided into two groups based on their coordination modes (Figure S1) according to the Harris notation: group A (with crystallographic residua _1, _2, and _3) displays a 5.22111 coordination mode, and the second group B (crystallographic residua _4, _5, and _6) has a 6.32121 coordination mode.^44^ Through all complexes, the SAA^3–^ ligands of group A differ only in the identity of the metal cation coordinated by the O3_*n* phosphonate oxygen atom (*n* = 1, 2, and 3). In complex **1**, this atom coordinates to Co^2+^ (Figure S2), and in complex **2**, this atom coordinates to Co^2+^ for *n* = 1 and to Na^+^ for *n* = 2 and 3 (Figure S3). In complex **3**, the O3_*n* atoms coordinate exclusively to Na^+^ (Figure S4). In group B, the differences lie in the metal cations coordinated to the O2_*n* and O4_*n* phosphonate oxygen atoms (*n* = 4, 5, 6). In complexes **1** and **3**, the O2_n and O4_*n* atoms coordinate exclusively to cobalt and sodium cations, respectively (Figures S2 and S4). In complex **2**, O2_4 and O4_4 coordinate sodium and cobalt cations, both O2_5 and O4_5 coordinate sodium cations, and both O2_6 and O4_6 coordinate cobalt and sodium, respectively (Figure S3).

The dysprosium cations, which occupy the central position in the molecular cores of **1**–**3**, are nine-coordinated, and the phosphonate oxygen atoms form their coordination spheres. The geometry analysis of the polyhedra by the Shape 2.1 program revealed the lowest shape parameter values of 1.538, 1.582, and 1.566 for **1**, **2**, and **3**, respectively, corresponding to the tricapped trigonal prismatic geometry (TCTPR-9) (Table S2).^45–47^ The cobalt cations, present in the complex core, are five-coordinated (Co1–3, Co7–9) and six-coordinated (Co4–6). The shape analysis of the coordination polyhedra revealed geometries close to the spherical square pyramidal (SPY-5) for all Co1–3 atoms (Table S3).^45,46,48,49^ The geometry of the polyhedron around the additional cobalt cation Co7 in **2** is close to a spherical square pyramid (SPY-5) with a shape parameter value equal to 2.223 (Table S3). The geometries of the polyhedra around the five-coordinated cobalt cations of **1** situated in the molecular apexes were found to be a trigonal bipyramid (TBPY-5) for disordered Co7 (occupancy of 0.22(3)) and Co7A (occupancy of 0.78(3)) with distortion values of 3.700 and 2.741, respectively, and a spherical square pyramid (SPY-5) for Co8 with a distortion value of 2.676. The chlorine atom around the Co9 atom is disordered and could have 2 positions depicted as Cl3 (occupancy: 0.858(6)) and Cl4 (occupancy: 0.142(6)). The coordination polyhedron around the Co9 atom with the most probable Cl3 atom in the coordination sphere is closer to a spherical square pyramid (SPY-5) with a distortion value of 2.085, while, with the Cl4 atom in the coordination sphere, it is closer to a trigonal bipyramid (TBPY-5) with a distortion value of 4.253 (Table S3). The coordination environment around Co4–6 atoms is closer to octahedral in all complexes, with distortion values ranging from 0.545 to 0.760. The bond distances in the polyhedra formed around the Co^2+^ and Dy^3+^ cations are listed in Table S4.

The formation of oxygen-bridged dinuclear Co_2_O_2_ units is observed in **1** and **2**. The Co4–Co9, Co5–Co7, and Co6–Co8 distances in **1** and the Co4–Co7 distance in **2** are approximately equal to 3.06 Å. These values are significantly lower than other Co–Co distances between the other neighboring cobalt atoms (∼3.6 Å), leading to a possible direct electronic and magnetic interaction between these cobalt atoms.

The significantly lower carbon content in the dried samples **1**–**3**, as revealed by the CHN elemental analysis, is clear evidence of the exchange of methanol molecules (coordinated and cocrystallized) for water during sample drying and storage (see Experimental Section).

All of the complexes and the disodium salt of the SAA^3–^ ligand were studied by IR spectroscopy. The spectra of the complexes are highly similar and differ from those of the ligand disodium salt due to coordination (Figure S5). But the main common feature throughout all spectra is the presence of intensive absorption bands, which are situated in the 800–1200 and 400–600 cm^–1^ regions and correspond to stretching and deformation vibrations of the —CPO_3_ group.^50,51^ Also, the ring C=C and imine C=N stretching vibrations could be found in the region of 1480–1660 cm^–1^.^52,53^ A low-intensity absorption band near 1300 cm^–1^ could correspond to the stretching vibration of the phenolic C–O bonds.^54^

Thermogravimetric analysis was conducted on complexes **1**–**3** to investigate their thermal properties (Figures S6–S8). The thermograms show similar behavior, starting with the endothermic evaporation of crystalline and coordinated solvent molecules. For complexes **1** and **3**, complete evaporation of the coordinated solvent molecules occurs at 286 and 225 °C, respectively. For complex **2** at 250 °C, most likely only the evaporation of crystalline water and partial evaporation of coordinated water molecules are observed (see Figure S7). The following exothermic mass losses correspond to the decomposition and oxidation of the organic groups. There is no mass change above 725 °C for complexes **1** and **3** and above 800 °C for complex **2** (Figures S6–S8).

Surprisingly, the obtained complexes **1**–**3** are well soluble in polar aprotic solvents, such as acetone, acetonitrile, and tetrahydrofuran (THF), in contrast to the phosphonates described previously.^23–25,55,56^ To investigate the behavior of complexes **1**–**3** in solution, ESI-MS spectrometry of their acetone solutions was conducted. The most intensive peaks in the ESI-MS spectra of the complexes **1**–**3** correspond to the [Co_9_Dy­(SAA)_6_Cl_3_ – Cl]^+^ (**1**), [Na_2_Co_7_Dy­(SAA)_6_(SA) + Na]^+^ (**2**), and [Na_3_Co_6_Dy­(SAA)_6_ + Na]^+^ (**3**) ions, and their isotopic patterns are in excellent agreement with calculated ones (Figure 3). The whole spectrum is presented in the Supporting Information (Figures S9–S11).

**Figure 3:**
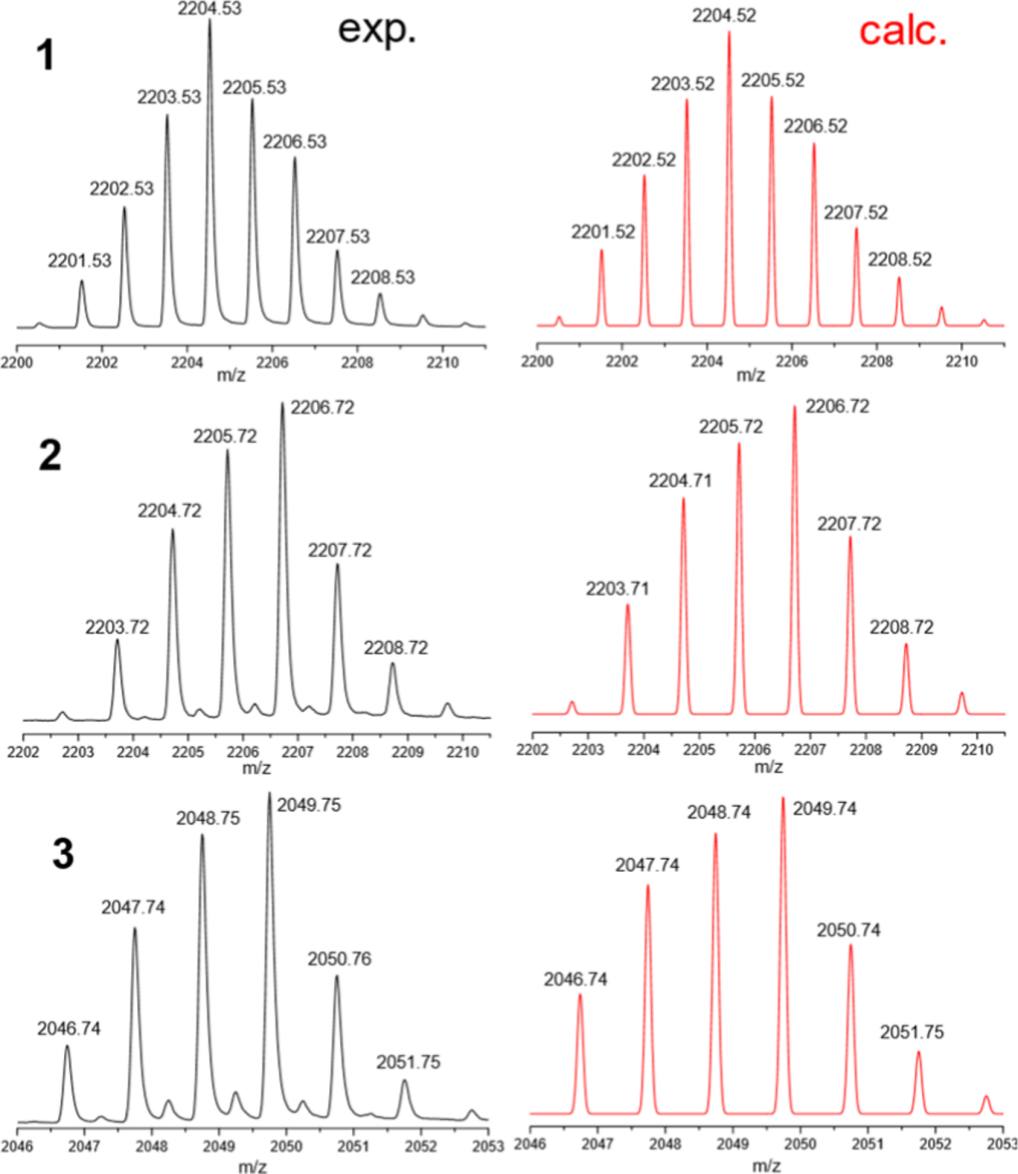
Comparison of the isotopic patterns of the most intensive peaks in ESI-MS spectra of complexes **1**–**3** (black) with the calculated ones (red).

This observation demonstrates the stability of the polynuclear molecular cores of **1**–**3** in the solution, which, in combination with the high solubility of these compounds in polar aprotic solvents and the presence of components which could be eliminated or substituted (MeOH, Cl^–^, or SA^–^), makes the molecules of **1**–**3** promising candidates as building blocks for further preparation of extended materials.

XPS analysis was employed to determine the oxidation state of cobalt in complexes **1**–**3** (Figure 4). The Co 2p_3/2_ peaks were observed at 781.7 eV (**1**) and 781.4 eV (**2** and **3**), while the Co 2p_1/2_ peaks appeared at 797.5 eV (**1**) and 797.2 eV (**2** and **3**), yielding a consistent spin–orbit splitting (Δ*E*_SO_) of 15.8 eV in all three samples. The position of the main Co 2p peak suggests the presence of Co^2+^.^57^ Moreover, the measured Δ*E*_SO_ values match the typical value of 16.0 eV for high-spin Co^2+^ species, confirming the presence of unpaired 3d electrons. These values point to the absence of Co^3+^ species.^58,59^ XPS spectra of C 1s were used for calibration and exhibit the presence of sp^3^, sp^2^, and sp carbon species.^60^

**Figure 4:**
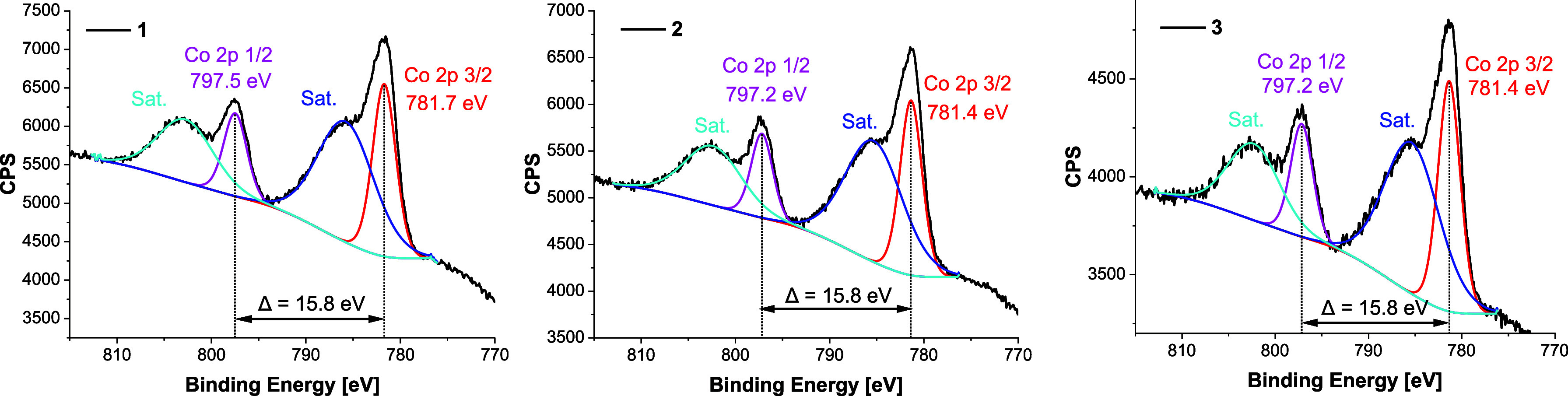
XPS spectra of Co 2p for complexes **1**–**3**.

Magnetic susceptibility data for complexes **1**–**3** were measured on polycrystalline samples over a temperature range of 2–300 K under a direct current magnetic field of 1000 Oe. The χ_m_ *T* versus *T* plots for these complexes are shown in Figure 5. At room temperature, the χ_m_ *T* values for complexes **1**–**3** are 39.68, 35.07, and 33.31 cm^3^ K mol^–1^, while the μ_eff_/μ_B_ values are 17.82, 16.75, and 16.32 (Figure S12), respectively. These values are approximately 17% higher than the theoretical ones calculated for the respective systems with uncoupled spins, involving Co^2+^, *g* = 2.0, *S* = 3/2, and Dy^3+^, *g*_*J*_ = 4/3, *J* = 15/2, which give the χ_m_ *T* values of 33.24, 29.23, and 27.22 cm^3^ K mol^–1^ and μ_eff_/μ_B_ values of 16.31, 15.29, and 14.76 for **1**–**3**, respectively. The slight increase in χ_m_ *T* values likely reflects differences in coordination and geometry among the three clusters (i.e., different metals and their environments), leading to distinct orbital contributions.

**Figure 5:**
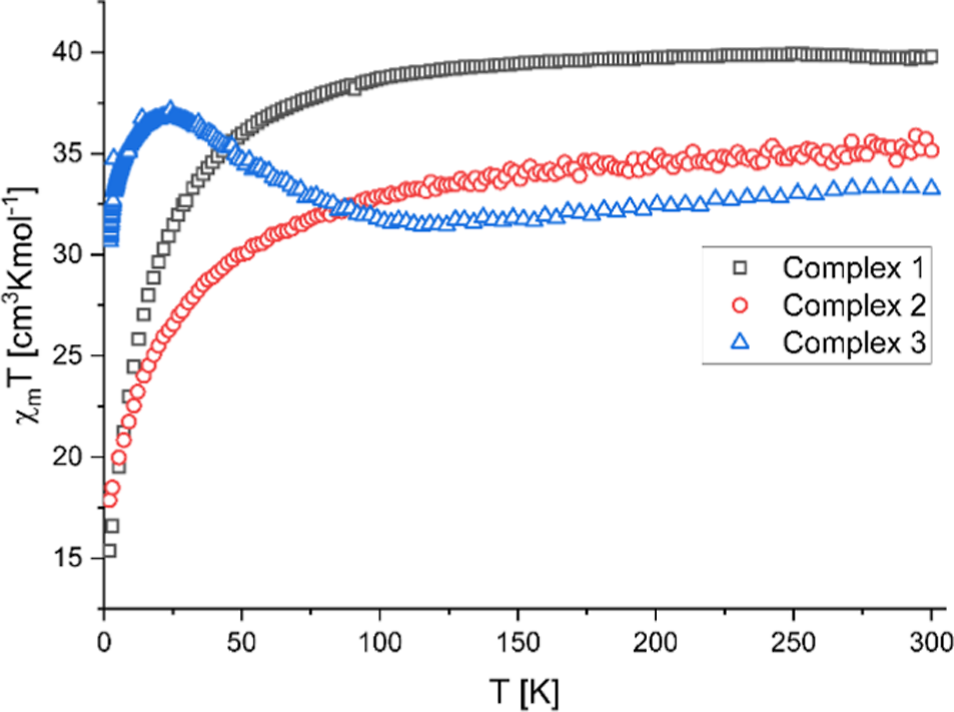
Temperature dependence of χ_m_T for complexes **1**–**3**, measured over the temperature range of 2–300 K under a dc field of 1000 Oe.

As the temperature decreases, the χ_m_ *T* and μ_eff_/μ_B_ values gradually decline to approximately 120 K in all of the complexes. However, only in the case of complexes **1** and **2** do the χ_m_ *T* values decrease more steeply, reaching the values of 15.36 cm^3^ K mol^–1^ and μ_eff_/μ_B_ = 11.08 for **1** and 17.87 cm^3^ K mol^–1^ and μ_eff_/μ_B_ = 11.95 for **2**, respectively, at 2 K. This behavior is consistent with the thermal depopulation of split energy levels of anisotropic Co^2+^ and Dy^3+^ ions at lower temperatures.^61^ Unlike complexes **1** and **2**, complex **3** exhibits a slight increase in χ_m_ *T* values below 120 K, reaching a maximum of 37.15 cm^3^ K mol^–1^ and μ_eff_/μ_B_ = 17.24 at 24 K. This behavior is consistent with the emergence of ferromagnetic correlations in an intermediate temperature regime. The subsequent pronounced decrease in χ_m_ *T* at lower temperatures can be rationalized by the strong magnetic anisotropy of the Dy^3+^ centers, spin–orbit coupling effects associated with Co^2+^, and possible additional insignificant antiferromagnetic or intermolecular contributions. Overall, the decrease in χ_m_ *T* for complexes **1**–**3** upon cooling can be attributed to a combination of factors, including antiferromagnetic interactions, strong magnetic anisotropy of the Dy^3+^ centers, and thermal depopulation of low-lying excited states associated with the Co^2+^ spin–orbit coupling. Consequently, the slightly negative Weiss constants obtained from Curie–Weiss fits (Θ ≈ from −0.4 to −2.6 cm^–1^, see Supporting Information, Figures S13–S15) in all cases should be regarded as effective parameters reflecting the overall magnetic response rather than unambiguous evidence for antiferromagnetic exchange.

To further evaluate the magnetic features of complexes **1**–**3**, the susceptibility data were analyzed with the PHI program (ver. 3.1.6).^62^ To keep the Hamiltonian tractable for a multinuclear 3d–4f cluster while retaining the dominant magnetic degrees of freedom, the Co^2+^ contribution was treated with effective spins, and a single isotropic g value was refined and shared across the Co^2+^ sites. The Dy^3+^ ion was described as an axial Kramers pseudospin-1/2 center, with an anisotropic *g* tensor (*g*⊥ = *g*_*x*_ = *g*_*y*_ and *g*∥ = *g*_*z*_). For complex **1** (Co_9_Dy), the fit of 1/χ_m_ = f­(*T*) is shown in Figure 6, while the fits of χ_m_ *T* = f­(*T*) and χ_m_ = f­(*T*) are depicted in Figure S16 in the Supporting Information. For complex **1**, the effective-spin analysis of the magnetic susceptibility data yielded an isotropic cobalt contribution with g_iso_(Co) = 2.17 and a strongly axial Dy^3+^ pseudospin description (*g*⊥ = *g*_*x*_ = *g*_*y*_ = 0.30 (fixed) and *g*∥ = *g*_*z*_ = 19.7), fully consistent with the Ising-like ground Kramers doublet commonly observed for Dy^3+^ ions, for which similar g_*z*_ values are reported.^63–67^ The fitted effective exchange parameters indicated weak antiferromagnetic interactions between the cobalt subsets (*J*_Co–Co_ = –0.34 cm^–1^) and a much smaller Co–Dy interaction (*J*_Co–Dy_ = – 0.09 cm^–1^). Previous studies on heterometallic Co–Dy systems have shown that magnetic behavior is predominantly governed by Dy^3+^ single-ion anisotropy, while 3d–4f exchange interactions typically play a secondary role.^68^ Overall, the monotonic decrease in χ_m_T upon cooling can therefore be attributed primarily to Dy^3+^ magnetic anisotropy and Co^2+^ spin–orbit coupling, with a negligible cooperative role for antiferromagnetic exchange.

**Figure 6:**
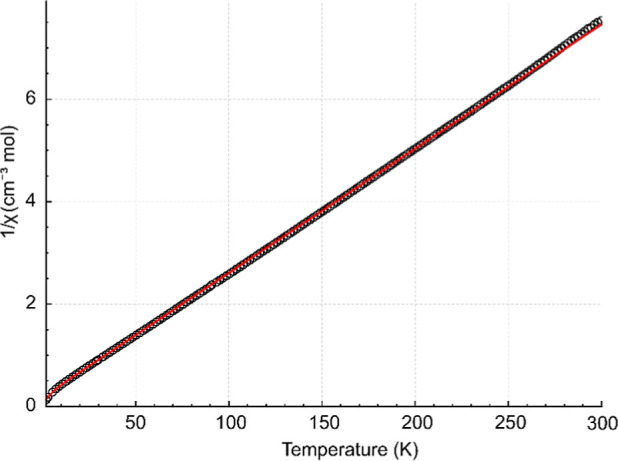
Temperature dependence (2–300 K) of 1/χ_m_ for complex **1**. The black circles represent the experimental data, and the red line describes the best-fit parameters.

The fit of 1/χ_m_ = f­(*T*) for the Co_7_Dy system (complex **2**) is shown in Figure 7, and the fits of χ_m_ *T* = f­(*T*) and χ_m_ = f­(*T*) are depicted in Figure S17 in the Supporting Information. Compared with the Co_9_Dy complex **1**, its Co_7_Dy analogue is characterized by a slightly smaller isotropic Co^2+^ response (g_iso_ = 2.05), while the Dy^3+^ center in Co_7_Dy (**2**) displays a more strongly axial ground doublet (*g*⊥ = 0.30 = fixed, *g*∥ = 22.00), which is consistent with a susceptibility profile dominated by single-ion anisotropy and spin–orbit coupling effects within the applied effective-spin description. The effective exchange analysis revealed slightly stronger antiferromagnetic Co–Co interactions in Co_7_Dy (**2**) (*J*_Co–Co_ = –0.72 cm^–1^) than in Co_9_Dy (**1**) (*J*_Co–Co_ = –0.34 cm^–1^), whereas the Co–Dy coupling remained very small in both cases and is close to zero in Co_7_Dy (**2**) (*J*_Co–Dy_ = +0.05 cm^–1^) compared to that in Co_9_Dy (**1**) (*J*_Co–Dy_ = –0.09 cm^–1^). Overall, increasing nuclearity from Co_7_Dy to Co_9_Dy is accompanied by a reduction in effective Co–Co antiferromagnetic interactions and a slightly less axial Dy^3+^ response, while the magnetic behavior in both systems remains dominated by strong single-ion anisotropy of Dy^3+^ and spin–orbit effects of Co^2+^.

**Figure 7:**
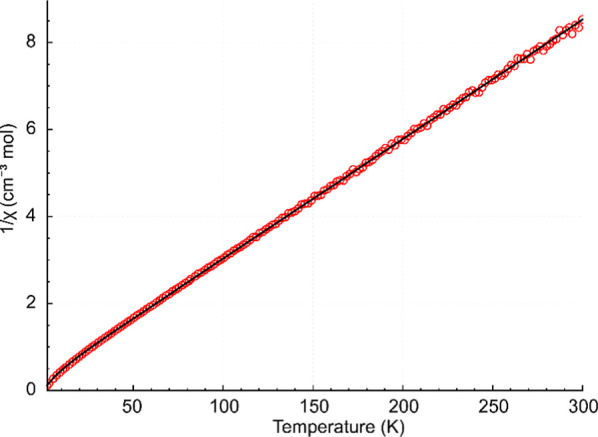
Temperature dependence (2–300 K) of 1/χ_m_ for complex **2**. The red circles represent the experimental data, while the black line describes the best-fit parameters.

Regarding complex **3** (a Co_6_Dy system), the initial attempts to interpret the experimental data over the full temperature range (2–300 K) were unsuccessful. In particular, the simplified effective Hamiltonian failed to simultaneously account for the pronounced low-temperature deviations (below 100 K) and the high-temperature Curie–Weiss-like behavior. This limitation can be attributed to the presence of additional low-energy effects at low temperatures such as short-range magnetic correlations, low-lying excited crystal-field states, or anisotropic exchange interactions, which are not explicitly included in the present effective model. The fitting range was therefore intentionally restricted to the high-temperature region (100–300 K) to avoid overparameterization and ensure a physically meaningful extraction of effective magnetic parameters. In this temperature range, the thermal population of higher-energy states suppresses low-energy correlations, allowing the magnetic susceptibility to be reliably described by effective Curie–Weiss-type behavior. The fit of the magnetic susceptibility data in the temperature range 100–300 K resulted in a good agreement between experimental and calculated curves (see Figures 8 and S18 in the Supporting Information). The best-fit parameters are as follows: *g*_iso_ = 2.0 (for Co^2+^ centers), *g*⊥ = 0.30 = fixed, *g*∥ = 17.5 (for the Dy^3+^ center), *z*J = –0.08 cm^–1^, and TI = –2.9 × 10^–4^ cm^3^ mol^–1^. The negative *z*J value indicated a very weak net antiferromagnetic mean-field contribution, most likely arising from intermolecular interactions. The small temperature-independent term (TI) accounts for residual Van Vleck-type contributions and minor systematic deviations of the model and should therefore be regarded as a phenomenological correction rather than a parameter derived from a specific microscopic interaction. Although the effective model adequately described the high-temperature (100–300 K) magnetic behavior, significant deviations observed below 100 K point to additional low-energy contributions not fully captured by the simplified Hamiltonian, potentially arising from low-lying crystal-field excitations or short-range magnetic correlations.

**Figure 8:**
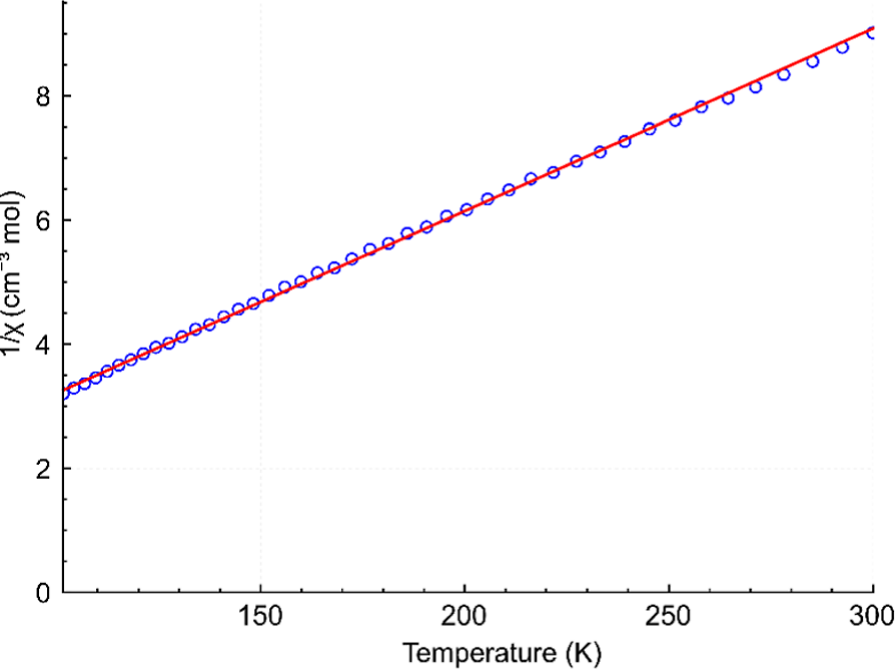
Temperature dependence (100–300 K) of 1/χ_m_ for complex **3**. The blue circles represent the experimental data, while the red lines describe the best-fit parameters.

The ac magnetic data were also measured for complexes **1**–**3**. Unfortunately, none of the complexes showed any significant frequency dependence of the out-of-phase susceptibility in ac experiments (see Figures S19–S21 in the Supporting Information). This indicates a lack of slow magnetization relaxation characteristic of SMM behavior.

## Conclusions

We report three new heterometallic Co–Dy phosphonate complexes: [Co_9_Dy­(SAA)_6_Cl_3_] (**1**), [Na_2_Co_7_Dy­(SAA)_6_(SA)] (**2**), and [Na_3_Co_6_Dy­(SAA)_6_] (**3**). Despite their different nuclearities, all of them share a common {Co_6_Dy} phosphonate core, while differences in the peripheral cations give rise to distinct {Co_9_Dy} (**1**), {Co_7_Dy} (**2**), and {Co_6_Dy} (**3**) architectures. Obtained complexes are notable for their unexpected solubility and stability in polar aprotic solvents, a feature rarely observed in phosphonate-based systems, thereby offering opportunities for exploration in homogeneous catalysis and enabling subsequent chemical transformations in solution. The magnetic behavior of the complexes is governed by the strongly axial Dy^3+^ ground Kramers doublet (*g*⊥ ≈ 0, large *g*∥), whereas the Co^2+^ centers are adequately described within an effective-spin approximation with moderate g values. Complexes **1** and **2** exhibited weak intracluster antiferromagnetic Co–Co coupling and negligible Co–Dy exchange, whereas complex **3** showed additional low-temperature contributions beyond the simplified effective model, although its high-temperature behavior remains anisotropy-dominated. Although none of the complexes exhibited slow magnetic relaxation typical of SMMs, the results highlight the sensitivity of 3d–4f exchange interactions to subtle structural changes. The modularity of the used ligand and the robustness of the {Co_*x*_Dy} phosphonate cores establish a promising platform for the systematic tuning of nuclearity, anisotropy, and exchange pathways.

Overall, our study extends the chemistry of heterometallic Co–Dy phosphonates and suggests straightforward strategies for designing other polynuclear 3d–4f assemblies. Their stability in solution makes them promising starting units for larger architectures with potential in magnetism and catalysis.

## Experimental Section

### General Procedures

All reactions were performed by using general synthetic techniques; no special conditions were used. Commercially available benzyl carbamate (TCI), phosphorus trichloride (Sigma), NaOH, salicylaldehyde (Sigma), methanol-*d*_4_ (Sigma), ethanol (p.a.), methanol (p.a.), acetone (p.a.), acetic acid (p.a.), acetonitrile (p.a.), DyCl_3_·6H_2_O (Sigma), and CoCl_2_·6H_2_O (Lachema) were used as received.

### Characterization Methods

The surface chemical composition was analyzed by X-ray photoelectron spectroscopy (XPS) on a Kratos Axis Supra device equipped with a monochromatic X-ray source with Al K_α_ (*E* = 1486.6 eV) excitation. The binding energy of 284.8 eV for C 1s was used for calibration. The high-resolution spectra were recorded with a 0.1 eV step. The deconvolution of the spectra was performed with the following parameters: peak shape GL(30), and the full width at half-maximum values of Co 2p3/2 and Co 2p1/2 were held equal. A Netsch STA 449 Jupiter instrument was used for thermogravimetric (TG) analyses. Samples were heated to 1000 °C in Pt crucibles at a heating rate of 5 °C min^–1^ in a synthetic air atmosphere with a flow rate of 100 cm^3^ min^–1^. Single-crystal X-ray diffraction measurements were performed on a Rigaku diffraction system (a MicroMax007HF DW rotating anode source with a multilayer optic, a partial χ*-axis* goniometer, a Saturn 724+ HG detector, and a Cryostream cooling device). The Mo–K_α_ (λ = 0.7107 Å) radiation was used. Data were corrected for Lorentz and polarization effects; absorption was taken into account on a semiempirical basis using multiple scans.^69–71^ *CrystalClear* (Rigaku 2014) and *CrysAlisPro* (Agilent Technologies 2013) software packages were used for data collection and reduction. The structures were solved using the *SHELXT* ^72^ program and refined (full matrix least-squares refinement on *F*_0_^2^) using the *SHELXL* ^73^ program. Elemental contents were measured by inductively coupled plasma optical emission spectroscopy (ICP-OES). ICP-OES analyses were done on an ICP-OES spectrometer iCAP PRO XPS Duo (Thermo, RF Power 1.10 kW, nebulizer gas flow 0.65 dm^3^ min^–1^, radial viewing height 11.0 mm). Elemental analysis (C, H, and N) was performed on a Flash 2000 CHNS Elemental Analyzer (Thermo Scientific). The ESI-MS spectra were measured on an Agilent 6224 Accurate-Mass TOF mass spectrometer (Agilent Technologies, Wilmington, DE, USA) with a dual electrospray ionization source from methanol solutions. The following parameters were used: nitrogen flow, 5 L min^–1^; gas temperature, 325 °C; nebulizer, 45 psi; skimmer, 65 V; and fragmentor, 50 and 100 V. IR spectra were recorded on a Bruker Tensor 27 Fourier transform infrared (FTIR) spectrometer with a Bruker Platinum ATR system. The solution NMR spectra were recorded on a Bruker Avance III 300 NMR spectrometer at frequencies of 300.1 MHz for ^1^H and 121.5 MHz for ^31^P in 5 mm NMR tubes. CD_3_OD was used as an internal lock. The spectra were referenced to the residual proton signal of CHD_2_OD (3.33 ppm), while the ^31^P spectra were referenced to 85% H_3_PO_4_ (0 ppm). The direct current (dc) magnetic susceptibility measurements (static magnetic data) were carried out on polycrystalline samples of complexes **1**–**3** over a temperature range of 2–300 K in an external magnetic field of 0.1 T using the MPMS Dynacool system (Quantum Design, San Diego, CA, USA) with a VSM option. The dynamic magnetic data (ac) were collected between 1.9 and 5.3 K under an external field *B*_dc_ = 0.1 T and a drive amplitude of 3.8 Oe within the 1–1488 Hz frequency range. All magnetic data were corrected for holder effects and diamagnetism. Magnetic susceptibility data were analyzed using the PHI program (version 3.1.6).^62^ The analysis was restricted to the temperature dependence of the molar magnetic susceptibility, χ­(*T*), together with χ*T*(*T*) and 1/χ­(*T*). A phenomenological effective-spin approach was used to ensure computational feasibility for multinuclear complexes **1**–**3**. Magnetically similar Co^2+^ centers were grouped into a limited number of effective subsets with a single isotropic g value refined for all Co sites. This treatment incorporates strong spin–orbit coupling effects and low-lying excited states into effective parameters without explicit orbital or crystal-field terms. Dy^3+^ centers were modeled as axial pseudospin-1/2 systems corresponding to their ground Kramers doublets with strongly anisotropic g tensors. Magnetic interactions were described using an effective spin Hamiltonian, including Zeeman and isotropic exchange terms. The exchange interaction (*J*) was defined according to the −2*J* convention implemented in the PHI program as

$${Ĥ}_{\mathrm{eff}}=-2\overset{i,j\in N}{\underset{i\neq j}{\sum }}J_{ij}\cdot {\mathrm{Ŝ⃗}}_{i}\cdot {\mathrm{Ŝ⃗}}_{j}+\mu_{B}\overset{N}{\underset{i}{\sum }}\mathrm{B⃗}\cdot g_{i}\cdot {\mathrm{Ŝ⃗}}_{i}$$

where *J*_ij_ represents effective exchange parameters between the defined spin subsets. Exchange interactions between the Co^2+^ subsets and between Co and Dy centers were treated at an effective level. The resulting exchange parameters should therefore be regarded as phenomenological quantities rather than microscopic superexchange constants.

### Ligand Synthesis

H_2_AIPA ((2-aminopropan-2-yl)­phosphonic acid monohydrate) was synthesized by using the literature method.^74^

The HSAA^2–^ ((2-{[(E)-(2-hydroxyphenyl)­methylidene]­amino}­propan-2-yl)­phosphonate) ligand was prepared by a Schiff base condensation reaction between H_2_AIPA and the corresponding aldehyde in the basic medium (NaOH) and isolated in the form of disodium salt monohydrate according to the previously used procedure.^25^

### Complex Synthesis

#### [Co_9_Dy­(SAA)_6_Cl_3_] (**1**)

The complex could be synthesized by three different methods:

##### Method A

A solution of DyCl_3_·6H_2_O (0.063 g, 0.17 mmol) in 10 cm^3^ of methanol was added to a stirred yellow solution of Na_2_HSAA·H_2_O (0.287 g, 0.941 mmol) with NaOH (1.00 mmol) in 30 cm^3^ of methanol. A solution of CoCl_2_·6H_2_O (0.357 g, 1.50 mmol) in 10 cm^3^ of methanol was added to the formed stirred solution. The clear solution turned brown, and the formed solution was filtered and left to stand in a closed flask. A large amount of a dark-green crystalline solid was observed the next day. The complex was left for crystallization for 1 week, and the solution became practically colorless. A few crystals were separated from the mother liquor for the single-crystal X-ray diffraction analysis; the rest of the product was separated by decantation, properly washed by methanol, and dried in open air, providing 0.312 g of [Co_9_Dy­(SAA)_6_Cl_3_(H_2_O)_4_(MeOH)_2_]·4H_2_O (yield 81.2% based on P, *M*_r_(C_62_H_90_Cl_3_Co_9_DyN_6_O_34_P_6_) = 2448.50 g mol^–1^).

Elemental analysis (Calcd for C_62_H_90_Cl_3_Co_9_DyN_6_O_34_P_6_/found): Dy 6.64/6.68; Co 21.66/21.60; P 7.59/7.59; C 30.41/30.42; H 3.70/3.31; N 3.43/3.60%.

TG-DSC (air): −8.7% endo (22–286 °C; 8.5% calcd for 8 H_2_O and 2 MeOH).

IR (cm^–1^): ν 3617 vw, 2977 vw, 1604 s, 1540 w, 1469 w, 1441 m, 1398 w, 1383 vw, 1366 vw, 1341 vw, 1313 w, 1286 w, 1210 w, 1115 vs, 1036 vs, 981 s, 937 m, 905 m, 851 w, 800 w, 752 m, 709 s, 632 w, 595 s, 553 vs, 539 vs, 498 w, 474 w, 446 s.

ESI-MS (positive ion mode, acetone): *m*/*z* 2204.53 [Co_9_Dy­(SAA)_6_Cl_3_ – Cl]^+^, 100%; 1084.78 [Co_9_Dy­(SAA)_6_Cl_3_ – 2 Cl]^2+^, 5.6%.

*Methods B* and *C* are described in the Supporting Information.

#### [Na_2_Co_7_Dy­(SAA)_6_(SA)] (**2**)

The complex could be synthesized by two different methods:

*Method A* is described in the Supporting Information.

##### Method B

As the first step, the complex [Co_7_(SAA)_2_(HSAA)_4_] was synthesized according to the procedure described in our previously published work^23^ by the reaction of CoCl_2_·6H_2_O (0.237 g, 1.00 mmol) and Na_2_HSAA·H_2_O (0.287 g, 0.941 mmol) in a methanol solution (30 cm^3^). The next day, DyCl_3_·6H_2_O (0.070 g, 0.185 mmol) was added directly to the reaction mixture, where a large amount of crystalline [Co_7_(SAA)_2_(HSAA)_4_] was formed. The mixture was stirred for 1 h, and NaOH (0.560 mmol) was added; the solution was stirred for the next 2 d. Slow dissolution of the complex [Co_7_(SAA)_2_(HSAA)_4_] and the color change from red-violet to light brown were observed. After the complex was dissolved, the mixture was filtered and left to stand in a closed flask, allowing the solvent to slowly evaporate. After evaporating half of the solvent, large brown crystals formed. The crystals were separated by decantation, washed with methanol, and dried in open air, providing 0.151 g of [Na_2_Co_7_Dy­(SAA)_6_(SA)­(H_2_O)_5_]·19H_2_O (yield 40.4% based on Co, *M*_r_(C_67_H_119_Co_7_DyN_6_Na_2_O_50_P_6_) = 2615.53 g mol^–1^).

Elemental analysis (Calcd for C_67_H_119_Co_7_DyN_6_Na_2_O_50_P_6_/found): Dy 6.21/6.19; Co 15.77/16.09; Na 1.76/1.98; P 7.11/7.34; C 30.77/30.47; H 4.59/4.49; N 3.21/3.31%.

TG-DSC (air): −14.6% endo (22–250 °C; 14.8% calcd for 21 H_2_O).

IR (cm^–1^): ν 3587 vw, 3347 w, 2972 w, 2931 w, 1614 s, 1538 m, 1467 m, 1442 s, 1396 m, 1363 vw, 1341 w, 1315 m, 1208 w, 1144 s, 1122 s, 1052 vs, 972 s, 921 m, 904 s, 852 m, 797 w, 756 s, 706 s, 631 s, 589 vs, 543 vs, 521 vs, 478 s, 442 vs.

ESI-MS (positive ion mode, acetone): *m*/*z* 2206.73 [Na_2_Co_7_Dy­(SAA)_6_(SA) + Na]^+^, 100.0%; 2062.71 [Na_2_Co_6_Dy­(SAA)_6_ + Co]^+^, 43.8%; 2049.75 [Na_3_Co_6_(SAA)_6_ + Na]^+^, 41.7%.

#### [Na_3_Co_6_Dy­(SAA)_6_] (**3**)

A solution of DyCl_3_·6H_2_O (0.063 g, 0.167 mmol) in 5 cm^3^ of methanol was added to a stirred yellow solution of Na_2_HSAA·H_2_O (0.287 g, 0.941 mmol) with NaOH (1.00 mmol). A fast-forming, flaky precipitate that immediately dissolved was observed. A solution of CoCl_2_·6H_2_O (0.238 g, 1.00 mmol) in 10 cm^3^ methanol was added to the formed stirred solution. The color of the clear solution changed from yellow to red-brown. The solution was concentrated by solvent evaporation on a rotary evaporator to a volume of 15 cm^3^. Some NaCl was observed to have formed. The solution was filtered and left to stand in a closed vial. After 1 h, large brown crystals started to form. After several days, crystals were collected by decantation, and several were left in the mother liquor for single-crystal X-ray diffraction analysis. Collected crystals were washed with methanol and dried in open air, providing 0.163 g of a powder product (crystals disintegrated on drying) [Na_3_Co_6_Dy­(SAA)_6_(H_2_O)_3_]·9H_2_O (yield 46.35% based on P, *M*_r_(C_60_H_90_Co_6_DyN_6_Na_3_O_36_P_6_) = 2242.29 g mol^–1^).

Elemental analysis (calcd for C_60_H_90_Co_6_DyN_6_Na_3_O_36_P_6_/found): Dy 7.25/7.04; Co 15.77/16.2; Na 3.08/3.06; P 8.29/8.08; C 32.14/32.22; H 4.05/3.82; N 3.75/3.76%.

TG-DSC (air): −9.9% endo (22–225 °C; 9.6% calcd for 12 H_2_O).

IR (cm^–1^): ν 3617 vw, 2971 w, 2930 w, 1612 s, 1537 m, 1467 m, 1442 m, 1395 w, 1362 vw, 1340 w, 1309 w, 1207 w, 1143 s, 1124 m, 1054 s, 1005 m, 962 s, 934 s, 904 s, 852 m, 794 w, 753 s, 739 m, 705 s, 630 s, 588 vs, 542 vs, 520 vs, 493 s, 476 s, 448 s.

ESI-MS (positive ion mode, acetone): *m*/*z* 2062.71 [Na_2_Co_6_Dy­(SAA)_6_ + Co]^+^, 9.4%; 2049.75 [Na_3_Co_6_Dy­(SAA)_6_ + Na]^+^, 100%; 2027.77 [Na_3_Co_6_Dy­(SAA)_6_ + H]^+^, 10.9%; 1036.37 [Na_3_Co_6_Dy­(SAA)_6_ + 2 Na]^2+^, 12.5%.

## Supplementary Material



## Notes

Additional synthetic methods of complexes **1** and **2** and their characterization (elemental analysis and IR); figures not included in the main text: structure clarification, IR, TG, ESI-MS, temperature dependence of the effective magnetic moment of the prepared complexes, Curie–Weiss analysis, and frequency dependence of the out-of-phase part of the ac susceptibility; and tables of the CShM analysis of the polyhedra of the prepared complexes and selected bond lengths (PDF)

## Notes

Iaroslav Doroshenko—Department of Chemistry, Masaryk University, Kotlarska 2, CZ-61137 Brno, Czech Republic; Tomas Pokorny—Department of Chemistry, Masaryk University, Kotlarska 2, CZ-61137 Brno, Czech Republic; Lucie Simonikova—Department of Chemistry, Masaryk University, Kotlarska 2, CZ-61137 Brno, Czech Republic; Zdenek Moravec—Department of Chemistry, Masaryk University, Kotlarska 2, CZ-61137 Brno, Czech Republic; Jaromír Marek—Department of Chemistry, Masaryk University, Kotlarska 2, CZ-61137 Brno, Czech Republic; Ján Vančo—Regional Center of Advanced Technologies and Materials, Czech Advanced Technology and Research Institute, Palacký University, Šlechtitelů 27, CZ-779 00 Olomouc, Czech Republic; Zdeněk Trávníček—Regional Center of Advanced Technologies and Materials, Czech Advanced Technology and Research Institute, Palacký University, Šlechtitelů 27, CZ-779 00 Olomouc, Czech Republic; Jiri Pinkas—Department of Chemistry, Masaryk University, Kotlarska 2, CZ-61137 Brno, Czech Republic

## Notes

Prior to submission, a preprint version of this work was released.^75^

## Abbreviations

ac: alternating current
BVS: bond valence sum
CShM: continuous shape measures
dc: direct current
ESI-MS: electrospray ionization mass spectrometry
FID: flame ionization detector
IR: infrared spectroscopy
FTIR: Fourier transformation infrared spectroscopy
ATR: attenuated total reflectance
ICP-OES: inductively coupled plasma atomic emission spectroscopy
TG: thermogravimetry
XPS: X-ray photoelectron spectroscopy
