# Supplementary material for: A Series of Solution-Stable Heterometallic Molecular Phosphonates with {Co9Dy}, {Co7Dy}, and {Co6Dy} Cores
Source: Inorg Chem. 2026 May 8;65(20):11314–25. doi: 10.1021/acs.inorgchem.6c01194 (PMC13213902; doi:10.1021/acs.inorgchem.6c01194)
Supplement: Supplementary file 1 [file ic6c01194_si_001.pdf]

Supporting Information for:

A Series of Solution-Stable Heterometallic Molecular Phosphonates with {Co_9Dy}, {Co_7Dy}, and {Co_6Dy} cores

Iaroslav Doroshenko[a], Tomas Pokorny[a], Lucie Simonikova[a], Zdenek Moravec[a], Jaromír Marek[a], Ján Vančo[b], Zdeněk Trávníček[b]*, and Jiri Pinkas[a]*

[a] *Department of Chemistry, Faculty of Science, Masaryk University, Kotlarska 2, CZ-61137 Brno, Czech Republic*

[b] *Regional Center of Advanced Technologies and Materials, Czech Advanced Technology and Research Institute, Palacký University, Šlechtitelů 27, CZ-779 00 Olomouc, Czech Republic*

* Corresponding authors.

E-mail: zdenek.travnicek@upol.cz (Z. Travníček), jpinkas@chemi.muni.cz (J. Pinkas).

Alternative methods of complex synthesis

[$Co_9Dy(SAA)_6Cl_3$] (**1**)

Method B

A solution of $DyCl_3 \cdot 6H_2O$ (0.051 g, 0.13 mmol) in 1 cm^3 of methanol was added to a suspension of $[Co_7(SAA)_2(HSAA)_4] \cdot 5MeOH$ (0.202 g, 0.100 mmol) in acetone (20 cm^3). Stepwise dissolution of the initial complex was accompanied by the formation of a green colored solution. After standing overnight, the solvents were evaporated completely on a rotary evaporator. The formed green solid was treated with acetone (4 cm^3), resulting in the formation of a clear emerald-green solution. The solution was filtered and left to stand in a closed vial at a temperature of 0–5 ^oC. A small amount of green plate-like crystals was formed after 3–5 d of standing in a fridge. The crystals were collected by decantation, and several were left in the mother liquor for the single-crystal X-ray diffraction analysis. Collected crystals were washed with methanol and dried in open air, providing 0.016 g of $[Co_9Dy(SAA)_6Cl_3(H_2O)_3] \cdot 8H_2O$ (yield 3.9 % based on P, $M_r(C_{60}H_{88}Cl_3Co_9DyN_6O_{35}P_6)$ = 2438.46 g mol^{-1}).

Elemental analysis (Calcd for $C_{60}H_{88}Cl_3Co_9DyN_6O_{35}P_6$ / found): Dy 6.66/6.61; Co 21.75/21.80; P 7.62/7.50; C 29.55/29.89; H 3.64/3.31; N 3.45/3.49 %.

IR (cm^{-1}): ν 3617 vw, 2977 vw, 1604 s, 1540 w, 1469 w, 1441 m, 1398 w, 1383 vw, 1366 vw, 1341 vw, 1313 w, 1286 w, 1210 w, 1115 vs, 1036 vs, 981 s, 937 m, 905 m, 851 w, 800 w, 752 m, 709 s, 632 w, 595 s, 553 vs, 539 vs, 498 w, 474 w, 446 s.

Method C

H_2AIPA (0.942 g, 6.00 mmol) and NaOH (0.720 g, 1.80 mmol) were suspended in 70 cm^3 of methanol and refluxed with continuous stirring. The solution of $DyCl_3 \cdot 6H_2O$ (0.377 g, 1.00 mmol) and $CoCl_2 \cdot 6H_2O$ (2.142 g, 9.00 mmol) in 50 cm^3 of methanol was added after the initial solution began to boil. After the mixture became homogeneous, salicylaldehyde (SA) (0.733

g, 6.00 mmol) was added. The color of the formed mixture changed from pink-violet to gray-green, and an insoluble precipitate formed. The solution was refluxed for 2 d and then cooled to room temperature. The solvent was evaporated completely by a rotary evaporator, and the solid residue was dissolved in 20 cm^3 of acetone. The solution was separated from the undissolved NaCl by filtration, then evaporated to dryness. The solid product was then treated with approximately 250 cm^3 of methanol and refluxed overnight. After the solution was cooled to room temperature, gray-green precipitate was filtered off, washed with methanol, and dried in open air, providing 1.131 g of $[Co_9Dy(SAA)_6Cl_3(H_2O)_3]\cdot 5H_2O$ (yield 47.4 % based on Dy, $M_r(C_{60}H_{82}Cl_3Co_9DyN_6O_{32}P_6)$ = 2384.41g mol^{-1}).

Elemental analysis (Calcd for $C_{60}H_{82}Cl_3Co_9DyN_6O_{32}P_6$ / found): Dy 6.82/6.82; Co 22.24/21.80; P 7.79/7.70; C 30.22/30.32; H 3.47/3.17; N 3.52/3.58 %.

IR (cm^{-1}): ν 3617 vw, 2977 vw, 1604 s, 1540 w, 1469 w, 1441 m, 1398 w, 1383 vw, 1366 vw, 1341 vw, 1313 w, 1286 w, 1210 w, 1115 vs, 1036 vs, 981 s, 937 m, 905 m, 851 w, 800 w, 752 m, 709 s, 632 w, 595 s, 553 vs, 539 vs, 498 w, 474 w, 446 s.

$[Na_2Co_7Dy(SAA)_6(SA)]$ (**2**)

Method A

$DyCl_3\cdot 6H_2O$ (0.063 g, 0.167 mmol) was added to a solution of $Na_2HSAA\cdot H_2O$ (0.287 g, 0.941 mmol), salicylaldehyde (SA) (0.020 g, 0.017 mmol), and NaOH (1.16 mmol) in 15 cm^3 of methanol. The mixture was shaken, and $CoCl_2\cdot 6H_2O$ (0.278 g, 1.16 mmol) was added after the dysprosium salt dissolved. The mixture was shaken again until the cobalt salt fully dissolved. The clear red-brown solution was filtered and left to stand in a closed vial. The next day, large brown crystals were formed. After a week of crystallization, the solution became practically colorless. Crystals were separated by decantation, washed with methanol, and dried in open

air, providing 0.390 g of $[Na_2Co_7Dy(SAA)_6(SA)(H_2O)_5]\cdot 19H_2O$ (yield 95.1 % based on P, $M_r(C_{67}H_{119}Co_7DyN_6Na_2O_{50}P_6)$ = 2615.53 g mol^{-1}).

Elemental analysis (Calcd for $C_{67}H_{119}Co_7DyN_6Na_2O_{50}P_6$ / found): Dy 6.21/6.04; Co 15.77/16.36; Na 1.76/1.68; P 7.11/6.92; C 30.77/30.58; H 4.59/4.49; N 3.21/3.28 %.

IR (cm^{-1}): ν 3365 w, 2979 w, 2931 vw, 1615 s, 1539 m, 1469 m, 1442 s, 1398 m, 1365 vw, 1340 w, 1309 m, 1256 vw, 1210 w, 1195 w, 1147 s, 1126 s, 1052 vs, 973 s, 904 s, 852 m, 798 w, 758 s, 739 m, 706 s, 631 s, 590 vs, 546 vs, 521 vs, 479 s, 440 vs.

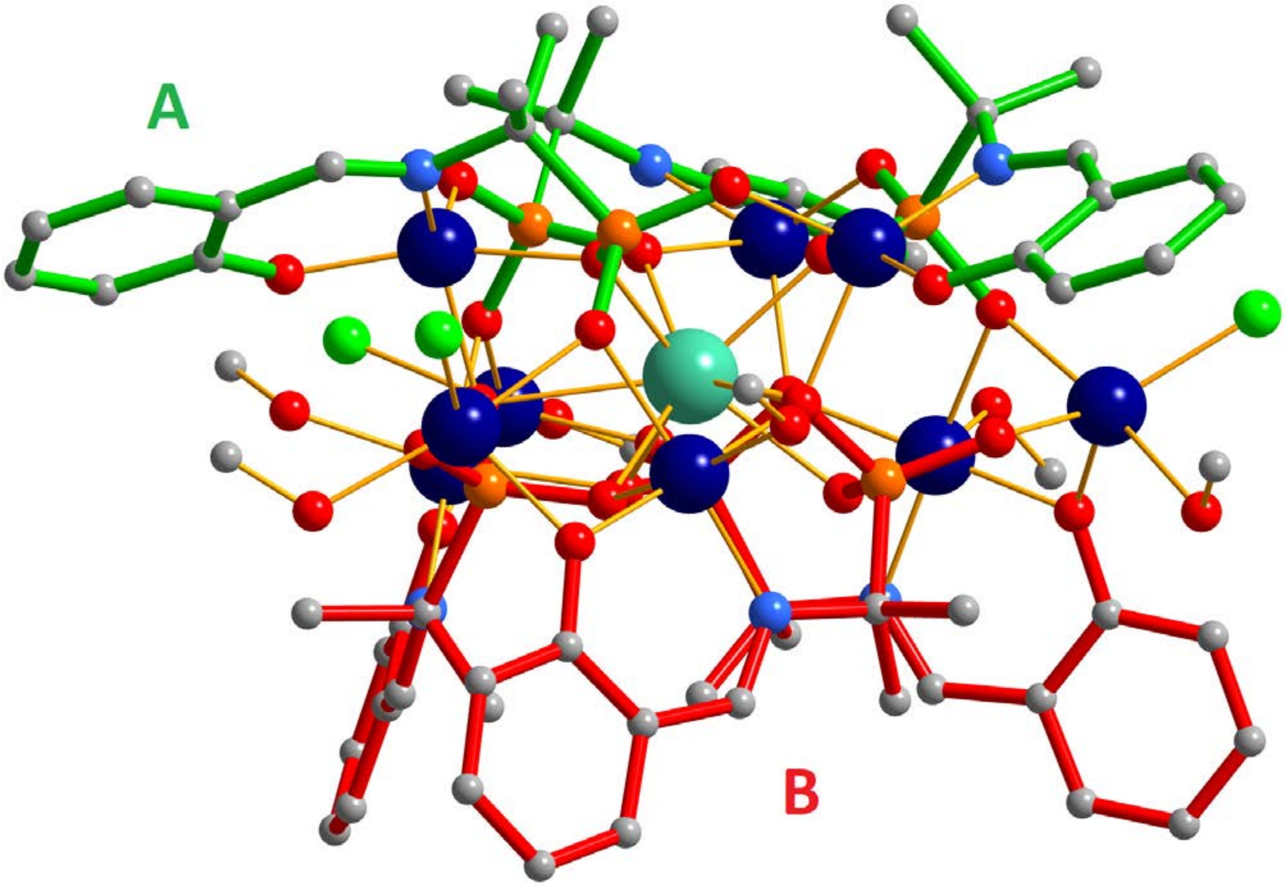

Figure S1. Ball and stick representation of two phosphonate ligand types (A and B) presented in complex molecules, on the example of complex **1**. The color code is the same as in **Figure 1** in the main text. All hydrogen atoms were omitted for the sake of clarity.

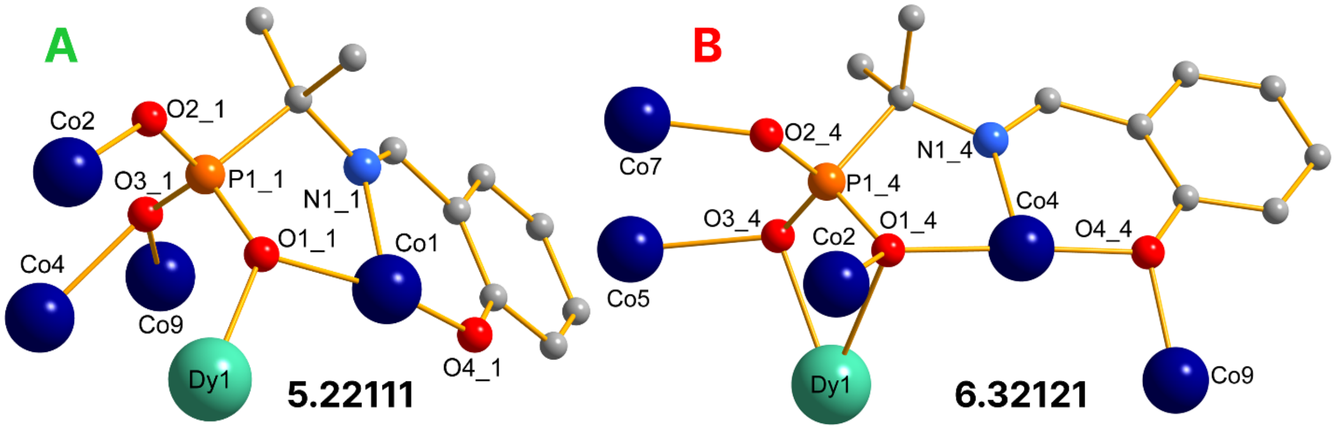

Figure S2. Ball and stick representation of the coordination modes of SAA^{3-} ligands, which are present in **1** with 5.22111 (type A) and 6.32121 (type B) coordination modes according to the Harris notation.[1] The color code is the same as in **Figure 1** in the main text. All hydrogen atoms were omitted for the sake of clarity.

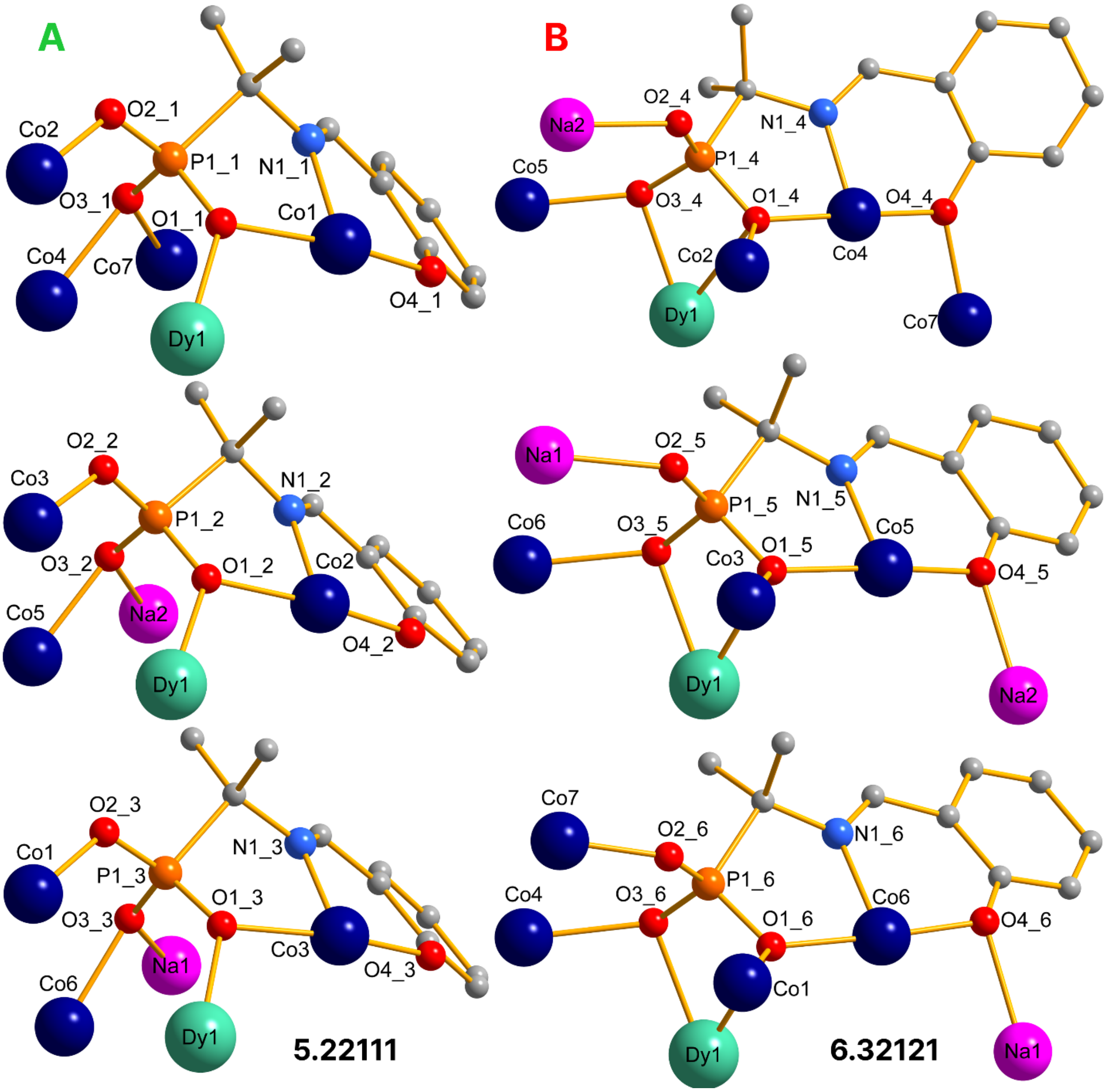

Figure S3. Ball and stick representation of the coordination modes of the SAA^{3-} ligands, which are present in **2** with 5.22111 (type A) and 6.32121 (type B) coordination modes according to the Harris notation.[1] The color code is the same as in **Figure 1** in the main text. All hydrogen atoms were omitted for the sake of clarity.

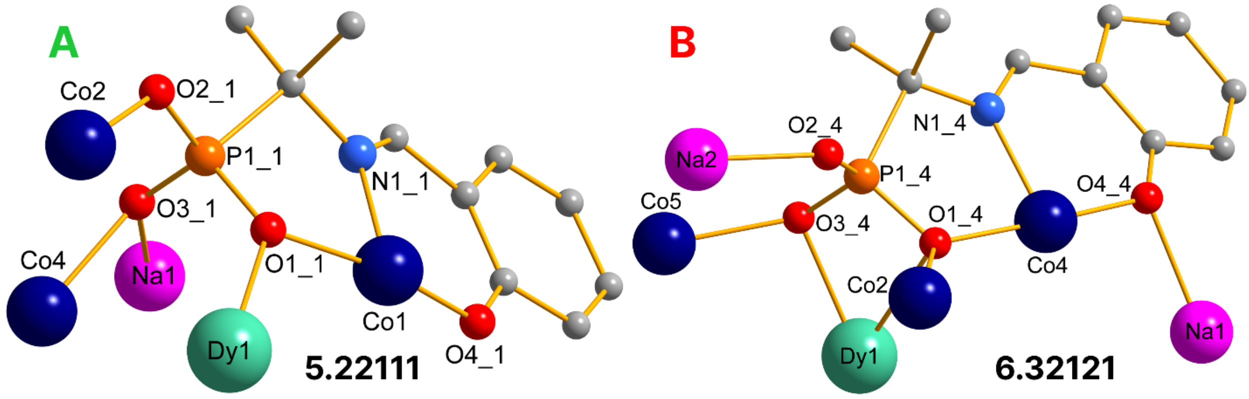

Figure S4. Ball and stick representation of the coordination modes of the SAA^{3-} ligands, which are present in **3** with 5.22111 (type A) and 6.32121 (type B) coordination modes according to the Harris notation.[1] The color code is the same as in **Figure 1** in the main text. All hydrogen atoms were omitted for the sake of clarity.

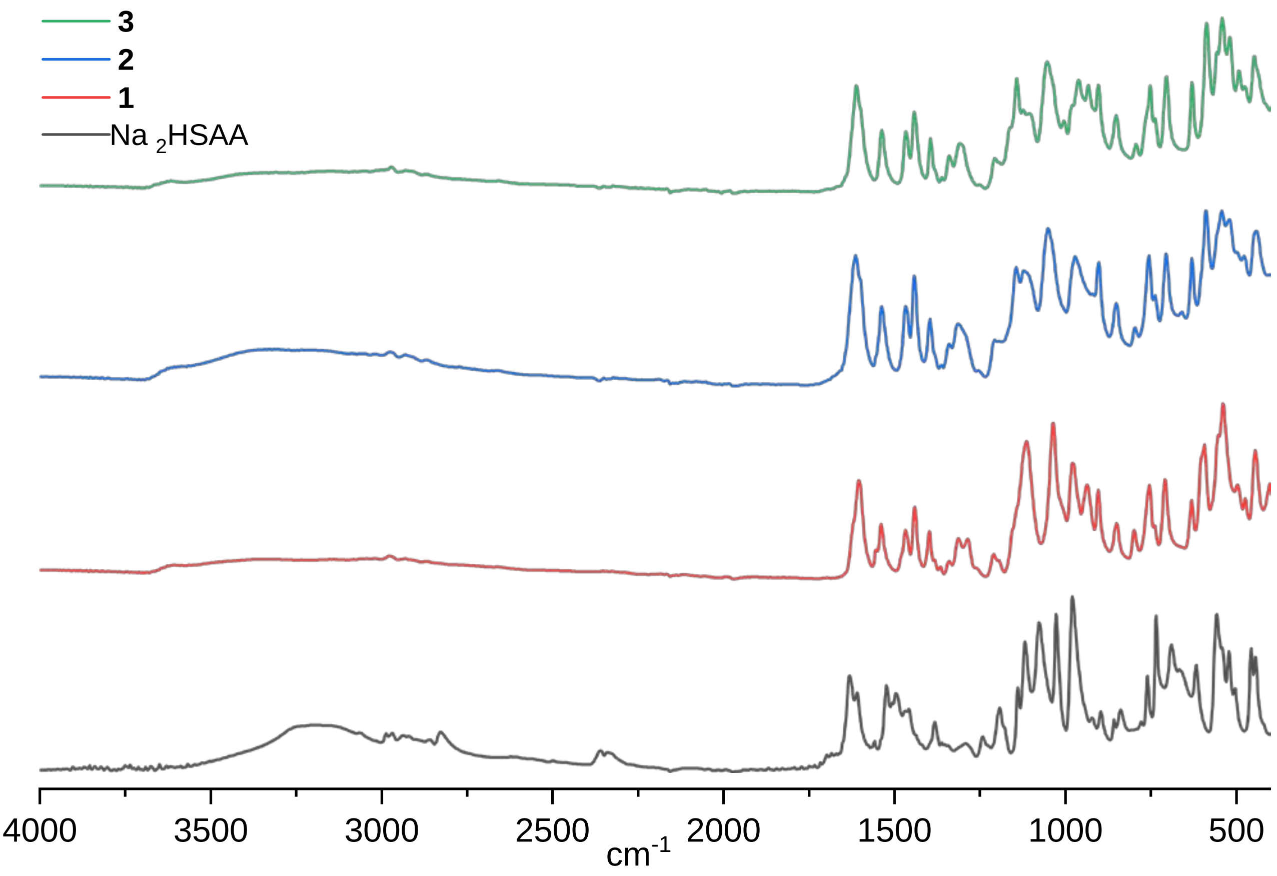

Figure S5. IR spectra of the ligand disodium salt (Na_2HSAA) and the complexes **1–3**.

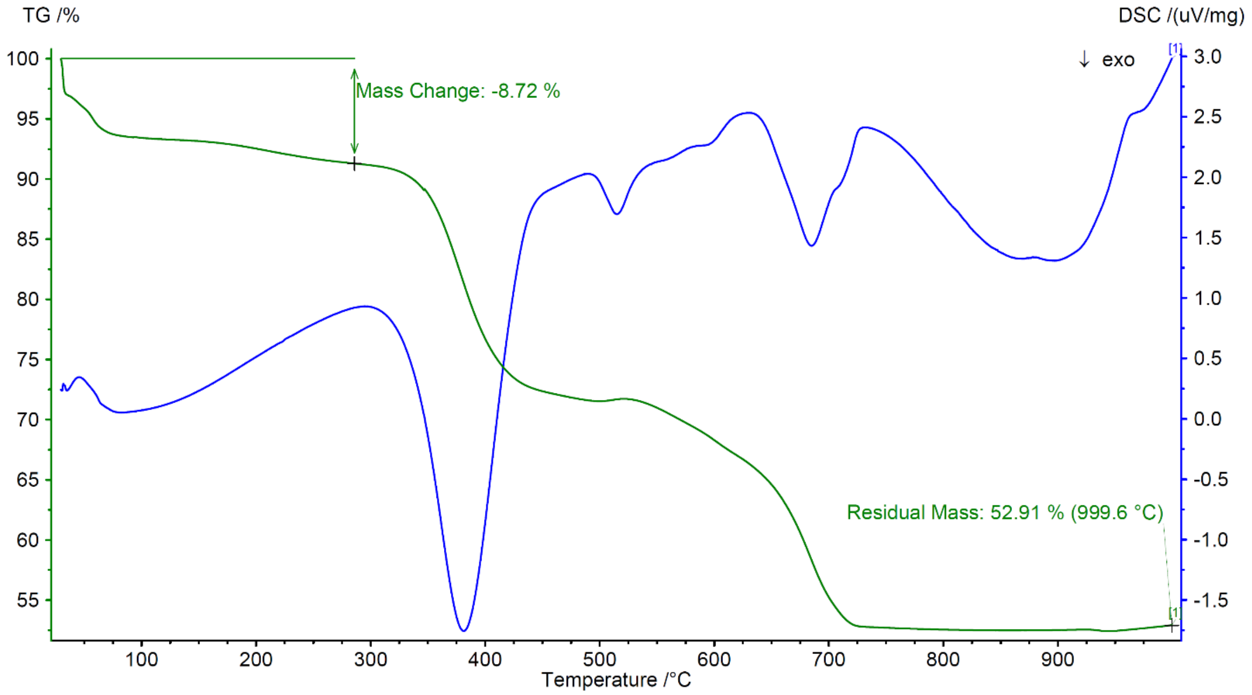

Figure S6. TG-DSC analysis of complex **1**.

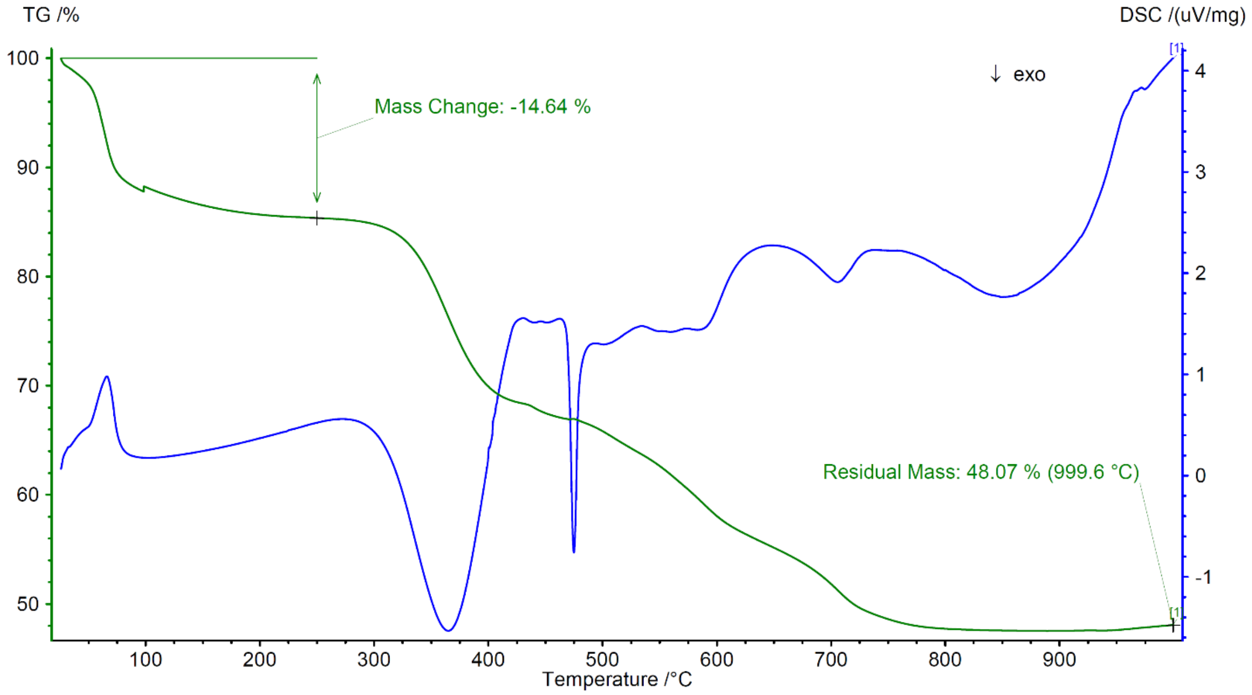

Figure S7. TG-DSC analysis of complex **2**.

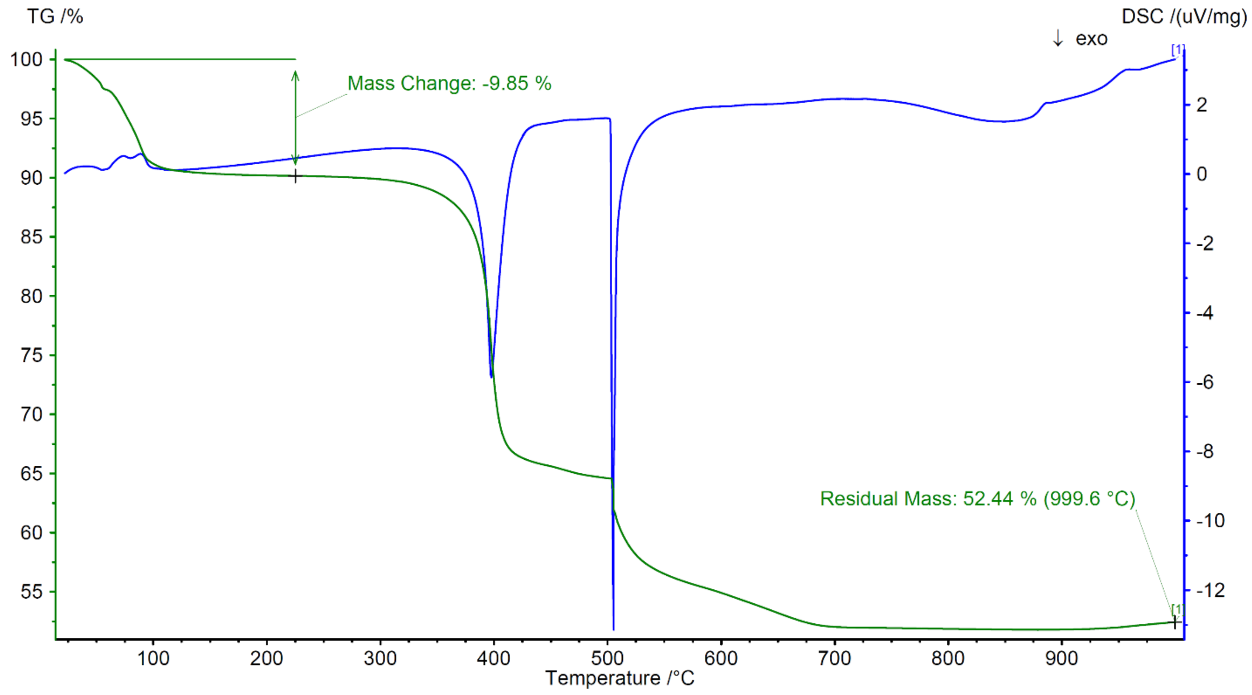

Figure S8. TG-DSC analysis of complex **3**.

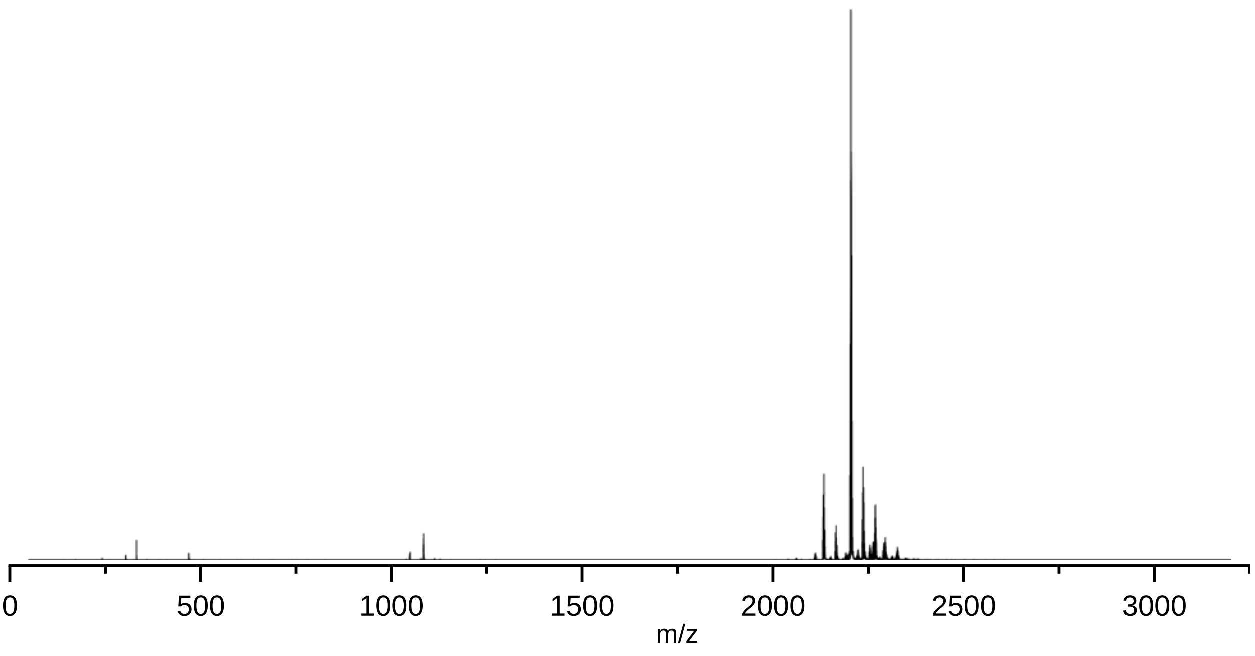

Figure S9. ESI-MS spectrum of complex **1** recorded in a positive ion mode.

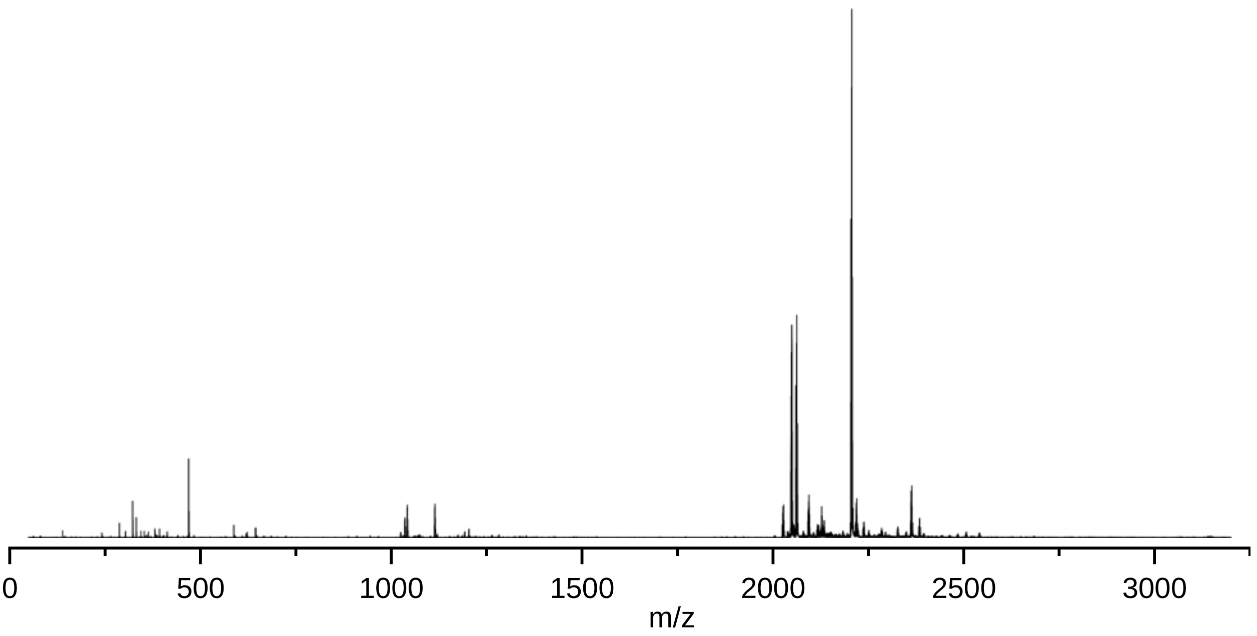

Figure S10. ESI-MS spectrum of complex **2** recorded in a positive ion mode.

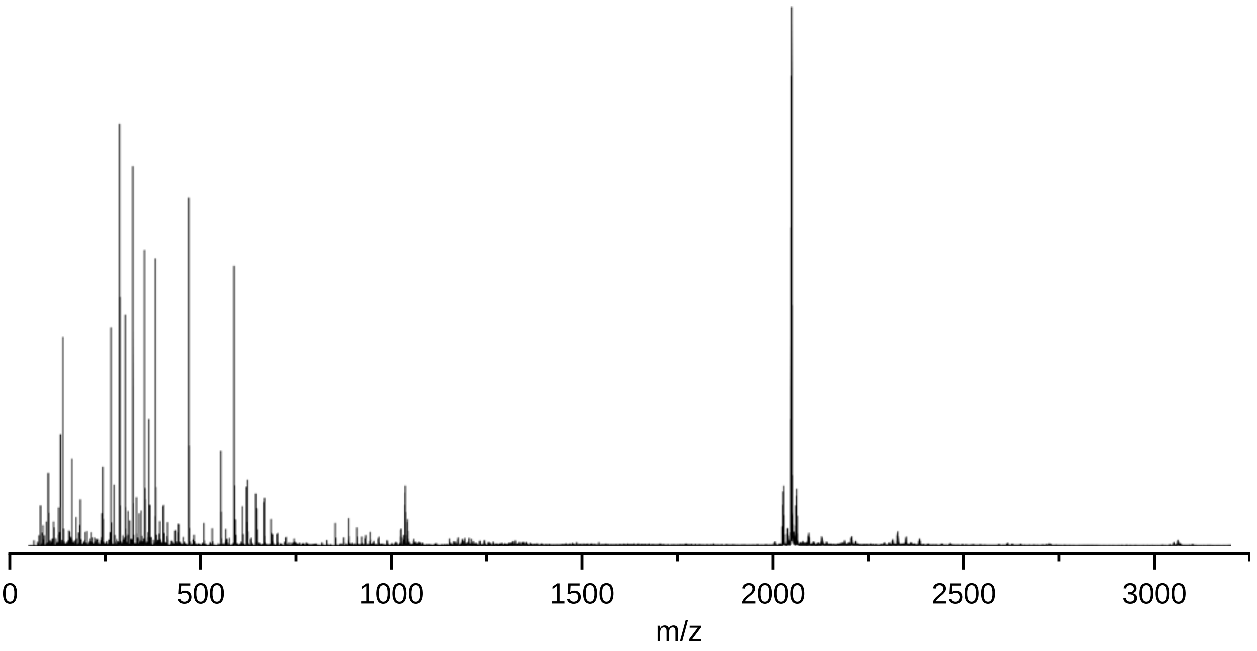

Figure S11. ESI-MS spectrum of complex **3** recorded in a positive ion mode.

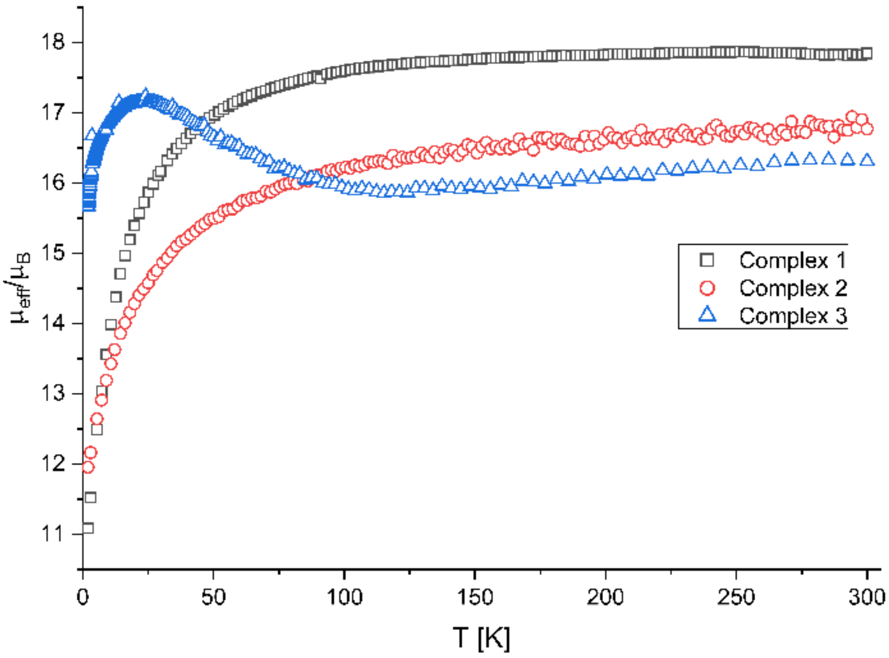

Figure S12. Temperature dependence of the effective magnetic moment μ_{eff}/μ_B for complexes **1–3**, measured over the temperature range of 2–300 K under the dc field of 1000 Oe.

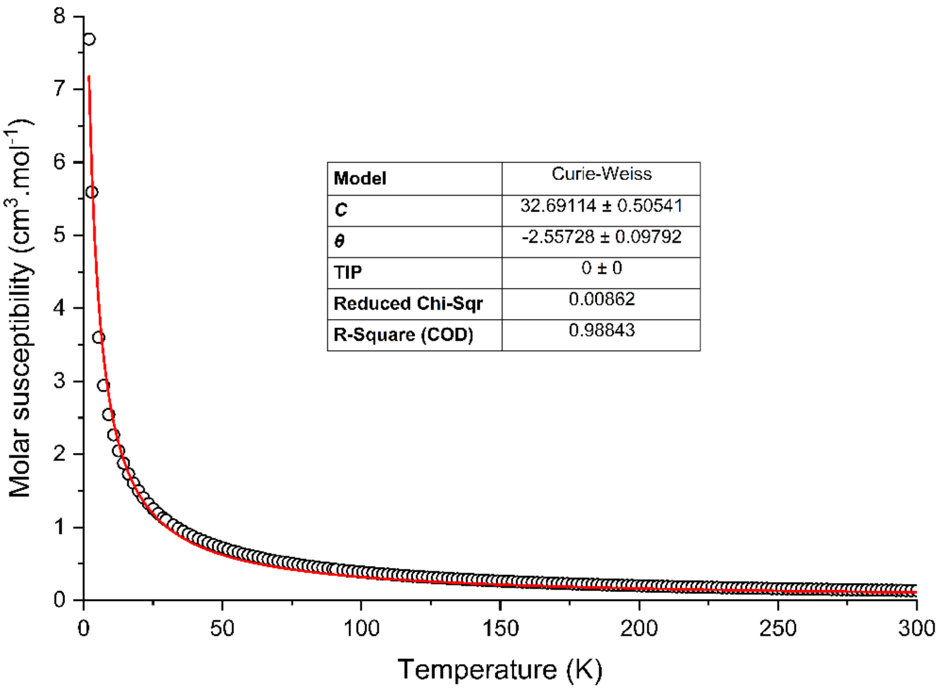

Figure S13. The Curie-Weiss analysis of magnetic susceptibility data for complex **1** (Co_9Dy). The inset shows the values of Curie and Weiss constants, along with statistics related to the fitting process (the red line indicates the best fit).

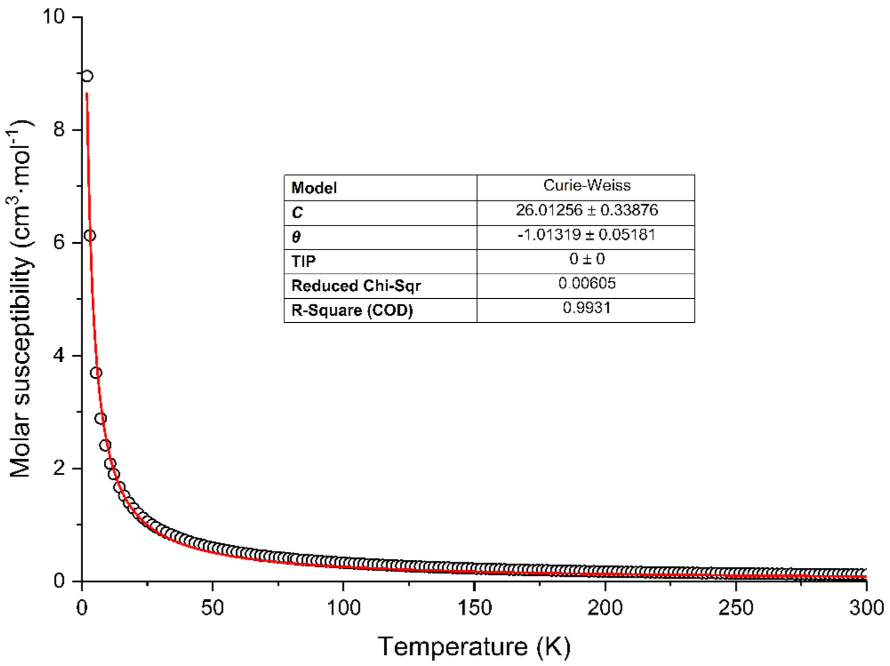

Figure S14. The Curie-Weiss analysis of magnetic susceptibility data for complex **2** (Co_7Dy). The inset shows the values of Curie and Weiss constants, along with statistics related to the fitting process (the red line indicates the best fit).

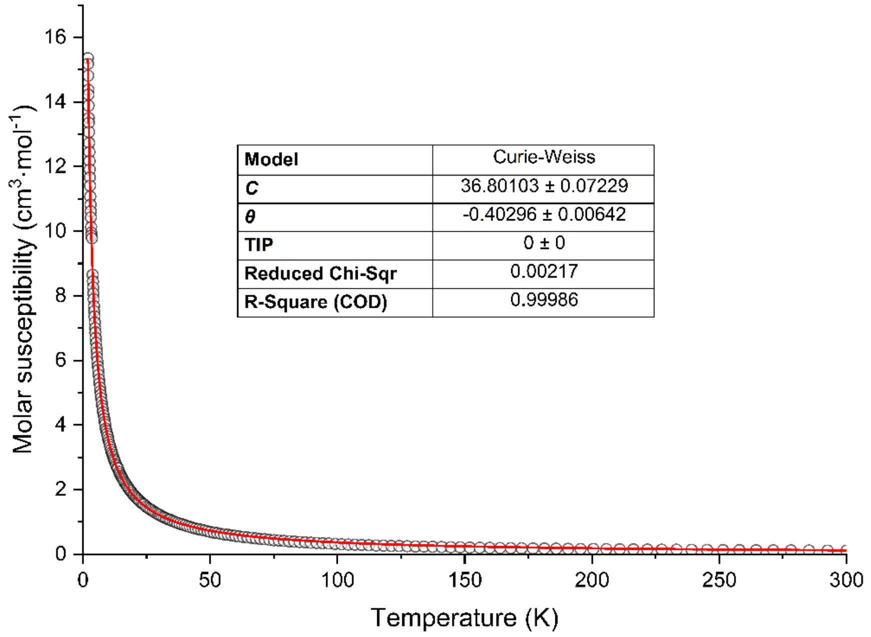

Figure S15. The Curie-Weiss analysis of magnetic susceptibility data for complex **3** (Co_6Dy). The inset shows the values of Curie and Weiss constants, along with statistics related to the fitting process (the red line indicates the best fit).

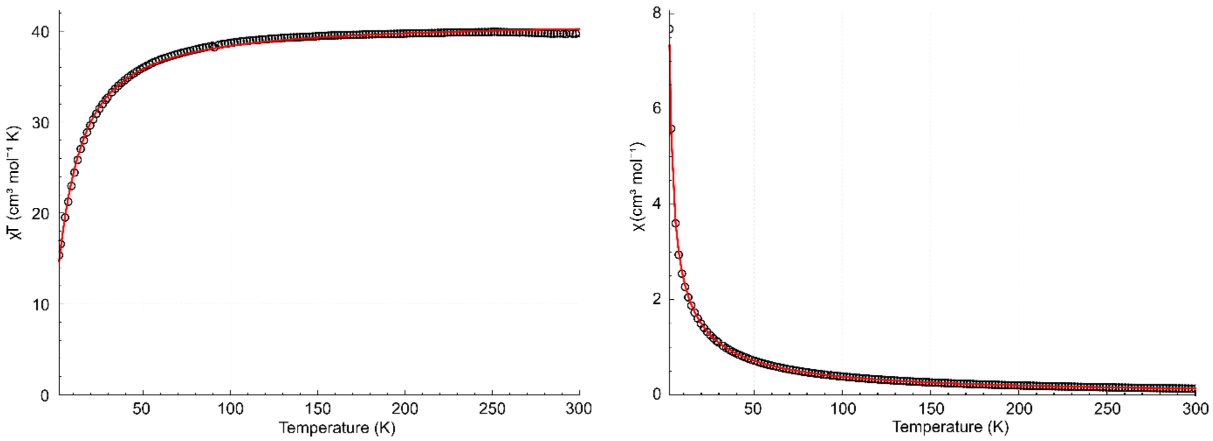

Figure S16. The fits of $\chi_m T = f(T)$ (*left*) and $\chi_m = f(T)$ (*right*) for complex **1** (Co_9Dy). The red line indicates the best fit.

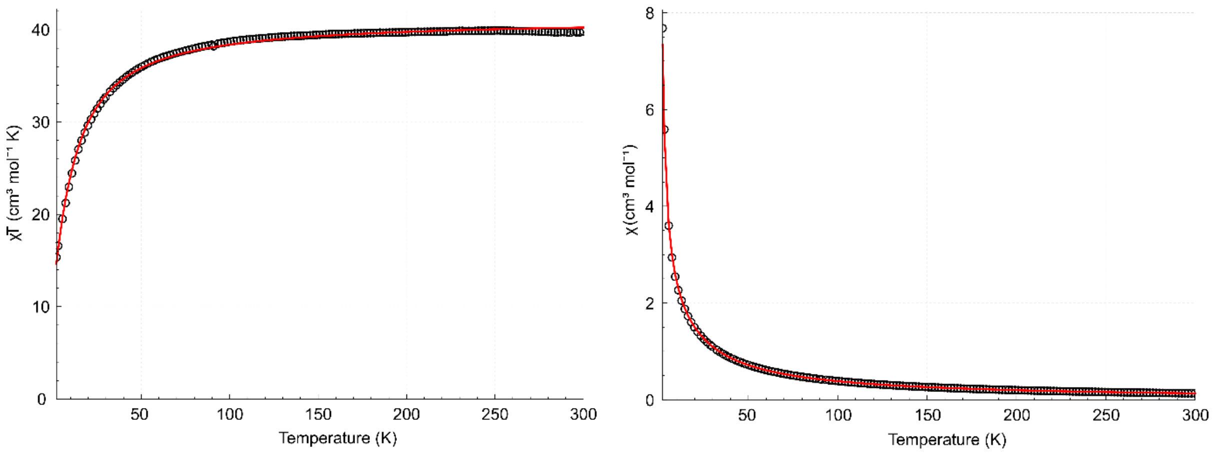

Figure S17. The fits of $\chi_m T = f(T)$ (*left*) and $\chi_m = f(T)$ (*right*) for complex **2** (Co_7Dy). The red line indicates the best fit.

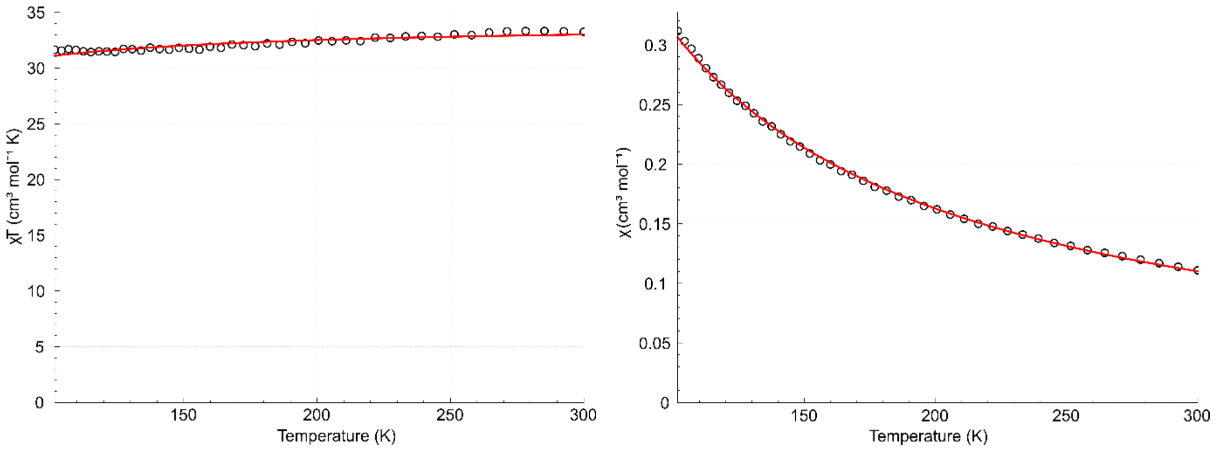

Figure S18. The fits of $\chi_m T = f(T)$ (*left*) and $\chi_m = f(T)$ (*right*) for complex **3** (Co_6Dy). The red line indicates the best fit.

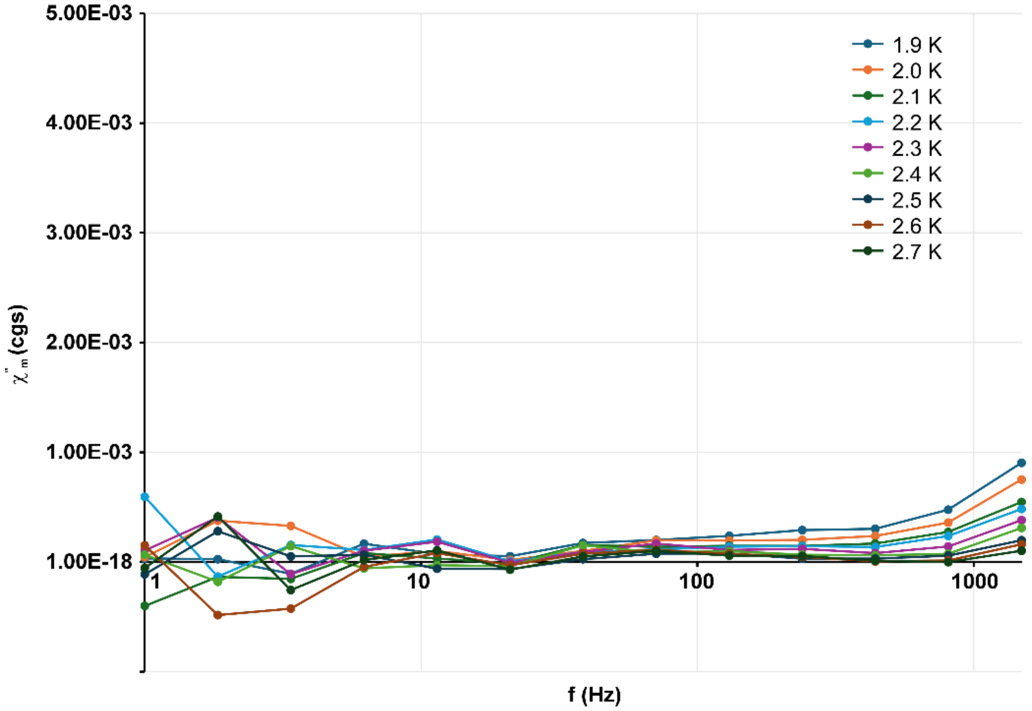

Figure S19. The frequency dependence of the out-of-phase part of the ac susceptibility (χ''_m) for **1** in the range of 1.9–2.7 K, showing a very weak signal, indicating that complex **1** cannot be regarded as a single molecule magnet (SMM).

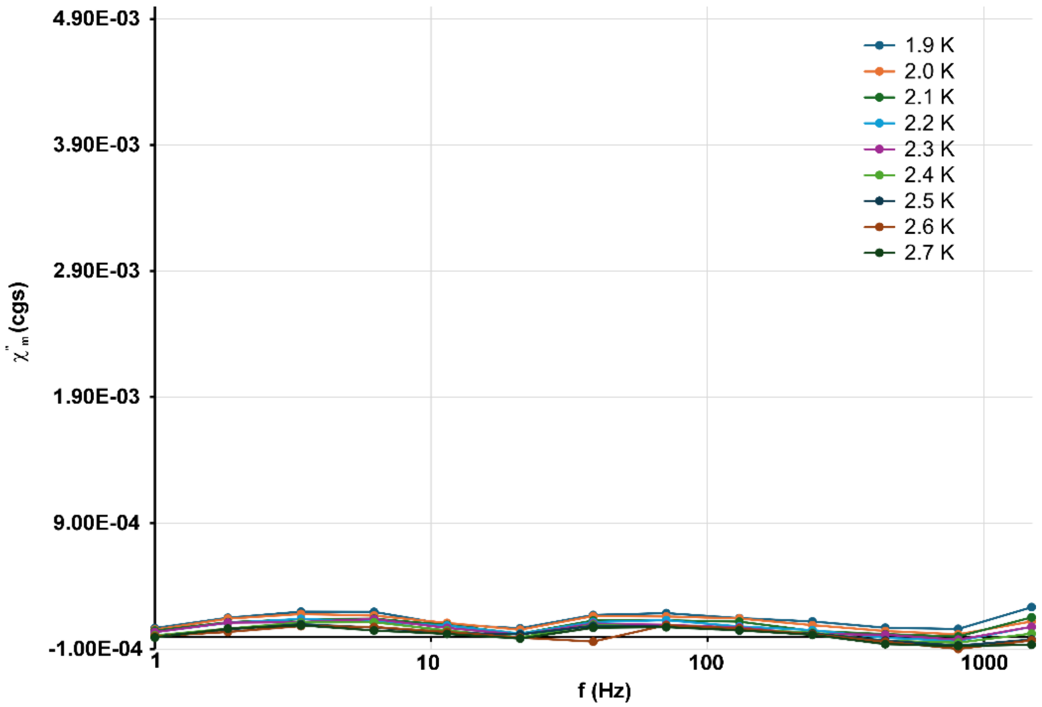

Figure S20. The frequency dependence of the out-of-phase part of the ac susceptibility (χ"$_m$) for **2** in the range of 1.9–2.7 K, showing a very weak signal, indicating that complex **2** cannot be regarded as a single molecule magnet (SMM).

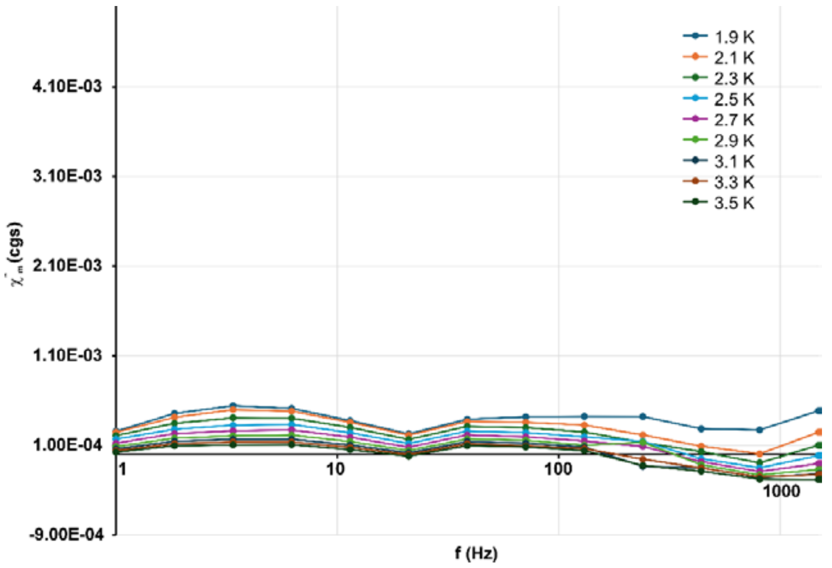

Figure S21. The frequency dependence of the out-of-phase part of the ac susceptibility (χ"$_m$) for **3** in the range of 1.9–3.5 K, showing a very weak signal, indicating that complex **3** cannot be regarded as a single molecule magnet (SMM).

Table S1. BVS calculated for the Dy and Co cations in **1**, **2**, and **3**.[2]

[a]Atom label	**1**	**2**	**3**
Dy1	2.99	3.00	2.98
Co1	1.96	1.89	1.98
Co2	1.97	1.98	1.98
Co3	1.92	1.92	1.98
Co4	2.11	2.11	2.02
Co5	2.07	2.04	2.05
Co6	2.13	1.98	2.04
Co7	2.25[b] / 1.97[c]	2.04	–
Co8	1.97	–	–
Co9	1.97 / 2.21[d]	–	–

[a] The bond valence sums (BVS) were calculated for the dysprosium and cobalt ions to establish their oxidation state Z_j from the bond valence S_{ij} using the following equations:

$$Z_j = \sum_i S_{ij}\ ;\ S_{ij} = \exp\left[\left(R_0 - r_{ij}\right)/b\right]$$

The parameter values for R_o equal 1.698, 1.720, 2.033, and 2.005 for Co–O, Co–N, Co–Cl, and Dy–O bonds, respectively. The usual value of 0.37 was used for b in both cases.

[b] BVS for Co7 was calculated with a coordination number value of 5; the value of 2.801 Å was used for Co7–O7 distance.

[c] The value for the disordered Co7A atom in **1**.

[d] The two values for disordered Cl3 / Cl4 atom in **1**.

Table S2. The geometry analysis of nine-vertex coordination polyhedra around the Dy1 atom in **1**, **2**, and **3** by the SHAPE 2.1 program.[3–5]

Complex	EP	OPY	HBPY	JTC	JCCU	CCU	JCSAPR
**1**	31.089	22.890	20.232	9.484	11.066	10.635	3.060
**2**	30.843	22.501	20.550	9.417	11.336	10.846	2.926
**3**	31.147	22.621	20.340	9.437	11.120	10.491	3.014

Complex	CSAPR	JTCTPR	TCTPR	JTDIC	HH	MFF
**1**	2.752	1.538	2.650	11.727	11.649	2.874
**2**	2.471	1.582	2.543	12.738	11.350	2.670
**3**	2.474	1.566	2.428	12.336	11.335	2.713

EP: Enneagon D_{9h}, OPY: Octagonal pyramid C_{8v}, HBPY: Heptagonal bipyramid D_{7h}, JTC: Johnson triangular cupola J3 C_{3v}, JCCU: Capped cube J8 C_{4v}, CCU: Spherical-relaxed capped cube C_{4v}, JCSAPR: Capped square antiprism J10 C_{4v}, CSAPR: Spherical capped square antiprism C_{4v}, JTCTPR: Tricapped trigonal prism J51 D_{3h}, TCTPR: Spherical tricapped trigonal prism D_{3h}, JTDIC: Tridiminished icosahedron J63 C_{3v}, HH: Hula-hoop C_{2v}, MFF: Muffin C_s.

Table S3. The geometry analysis of Co^{2+} polyhedra in **1**, **2**, and **3** by the SHAPE 2.1 program.[3,4,6,7]

Coordination number	Complex	Central atom	PP-5	vOC-5	TBPY-5	SPY-5	JTBPY-5
5	**1**	Co1	26.536	3.428	2.913	1.933	5.738
		Co2	27.143	3.104	3.235	1.588	5.775
		Co3	27.465	3.725	2.374	2.151	4.824
		Co7	28.762	5.459	3.700	4.731	4.835
		Co7A	28.053	4.161	2.741	3.782	3.888
		Co8	25.249	2.835	5.097	2.676	6.595
		Co9 (Cl3)	27.586	2.260	3.417	2.085	5.356
		Co9 (Cl4)	28.608	7.361	4.253	6.049	5.832
	**2**	Co1	27.272	2.333	3.430	1.516	5.505
		Co2	25.603	3.423	2.672	2.320	5.259
		Co3	26.616	2.827	3.127	1.647	5.676
		Co7	28.719	2.652	2.365	2.223	3.532
	**3**	Co1	28.877	2.110	2.971	1.235	5.569
		Co2	27.377	2.026	3.467	1.256	5.968
		Co3	29.107	2.074	3.074	1.108	5.619
			HP-6	PPY-6	OC-6	TPR-6	JPPY-6
6	**1**	Co4	31.120	26.569	0.545	14.127	30.321
		Co5	31.424	26.510	0.730	13.501	29.906
		Co6	31.492	25.621	0.760	12.895	29.519
	**2**	Co4	31.018	26.302	0.726	13.955	29.642
		Co5	30.750	26.008	0.619	13.235	29.323
		Co6	31.422	27.257	0.662	13.776	30.607
	**3**	Co4	31.685	26.492	0.632	14.062	30.535
		Co5	32.105	27.783	0.541	15.017	30.969
		Co6	31.416	27.764	0.653	13.821	31.417

PP-5: Pentagon D_{5h}; vOC-5: Vacant octahedron C_{4v}; TBPY-5: Trigonal bipyramid D_{3h}; SPY-5: Spherical square pyramid C_{4v}; JTBPY-5: Johnson trigonal bipyramid J12 D_{3h}; HP-6: Hexagon D_{6h}; PPY-6: Pentagonal pyramid C_{5v}; OC-6: Octahedron O_h; TPR-6: Trigonal prism D_{3h}; JPPY-6: Johnson pentagonal pyramid J2 C_{5v}.

Table S4. Bond lengths (Å) in Dy^{3+} and Co^{2+} polyhedra in **1**, **2**, and **3**.

Central atom	Donor atom	**1**	**2**	**3**
Dy	O1_1	2.281(3)	2.319(7)	2.3206(14)
	O1_2	2.290(3)	2.290(6)	2.2933(13)
	O1_3	2.301(3)	2.279(7)	2.2998(14)
	O1_4	2.600(3)	2.617(7)	2.5941(14)
	O1_5	2.680(3)	2.562(7)	2.5802(14)
	O1_6	2.673(3)	2.621(6)	2.6184(14)
	O3_4	2.406(3)	2.389(6)	2.4154(14)
	O3_5	2.353(3)	2.405(7)	2.3971(14)
	O3_6	2.399(3)	2.415(7)	2.3834(14)
Co1	O4_1	1.949(3)	1.994(7)	1.9603(15)
	O2_3	2.027(3)	2.009(8)	2.0322(15)
	N1_1	2.034(4)	2.026(10)	2.0249(17)
	O1_6	2.128(3)	2.117(7)	2.1049(14)
	O1_1	2.141(3)	2.211(6)	2.1320(13)
Co2	O4_2	1.952(3)	1.948(8)	1.9529(15)
	O2_1	1.982(3)	2.020(7)	2.0394(15)
	N1_2	2.031(4)	2.019(8)	2.0167(17)
	O1_4	2.124(3)	2.123(6)	2.1052(14)
	O1_2	2.190(3)	2.160(7)	2.1455(13)
Co3	O4_3	1.962(3)	1.962(8)	1.9529(16)
	O2_2	2.006(3)	2.034(7)	2.0252(14)
	N1_3	2.037(4)	2.038(8)	2.0226(19)
	O1_5	2.115(3)	2.116(6)	2.1027(15)
	O1_3	2.213(3)	2.174(8)	2.1513(14)
Co4	O4_4	1.995(3)	1.968(7)	1.9687(15)
	N1_4	2.041(3)	2.046(8)	2.0791(18)
	O1_4	2.068(3)	2.031(7)	2.0598(14)
	O5_4	2.095(3)	2.155(8)	2.1791(14)
	O3_1	2.173(3)	2.149(7)	2.1648(15)
	O3_6	2.199(3)	2.245(7)	2.2393(14)
Co5	O4_5	1.977(3)	1.969(7)	1.9700(16)
	O1_5	2.050(3)	2.108(6)	2.0663(14)
	N1_5	2.058(4)	2.056(9)	2.0694(18)
	O5_5	2.123(3)	2.168(8)	2.1675(17)
	O3_2	2.181(3)	2.169(7)	2.1811(15)
	O3_4	2.241(3)	2.198(7)	2.2091(14)
Co6	O4_6	1.965(3)	1.979(8)	1.9669(15)
	N1_6	2.045(4)	2.089(8)	2.0801(19)
	O1_6	2.059(3)	2.060(7)	2.0568(14)
	O5_6	2.129(3)	2.178(7)	2.1735(16)
	O3_3	2.150(3)	2.147(7)	2.1517(16)
	O3_5	2.208(3)	2.301(6)	2.2363(14)
Co7 / Co7A[c]	O2_4 (O2_6)[a]	2.140(13) / 1.930(6)	1.951(8)	
	O4_5 (O4_4)	1.921(11) / 2.044(5)	2.026(7)	
	O3_2 (O3_1)	1.76(3) / 2.266(14)	2.212(7)	
	Cl1 (O4_7)	2.289(17) / 2.213(3)	1.940(9)	
	O7 (O1_7)	- / 2.266(14)	2.081(8)	
Co8	O2_5	2.019(3)		
	O4_6	2.052(3)		
	O8	2.063(4)		
	O3_3	2.112(3)		
	Cl2	2.3178(18)		
Co9	O2_6	1.978(3)		
	O4_4	2.030(3)		
	O3_1	2.161(3)		
	O9	2.170(4)		
	Cl3	2.2677(14)		
	Cl4[b]	2.129(9)		

[a] In the brackets, donor atoms for complex **2**
[b] Disordered chlorine atom
[c] Co7A is disordered Co7 atom for complex **1**

References

(1) Coxall, R. A.; Harris, S. G.; Henderson, D. K.; Parsons, S.; Tasker, P. A.; Winpenny, R. E. P. P. Inter-Ligand Reactions: In Situ Formation of New Polydentate Ligands. *J. Chem. Soc. Dalt. Trans.* **2000**, *14* (14), 2349–2356. https://doi.org/10.1039/b001404o.

(2) Brown, I. D. *The Chemical Bond in Inorganic Chemistry: The Bond Valence Model*; Oxford University Press, 2002.

(3) Llunell, M.; Casanova, D.; Cirera, J.; Alemany, P.; Alvarez, S. *SHAPE 2.1*; Universitat de Barcelona: Barcelona, 2013.

(4) Pinsky, M.; Avnir, D. Continuous Symmetry Measures. 5. The Classical Polyhedra. *Inorg. Chem.* **1998**, *37* (21), 5575–5582. https://doi.org/10.1021/ic9804925.

(5) Ruiz-Martínez, A.; Casanova, D.; Alvarez, S. Polyhedral Structures with an Odd Number of Vertices: Nine-Coordinate Metal Compounds. *Chem. Eur. J.* **2008**, *14* (4), 1291–1303. https://doi.org/10.1002/chem.200701137.

(6) Alvarez, S.; Llunell, M. Continuous Symmetry Measures of Penta-Coordinate Molecules: Berry and Non-Berry Distortions of the Trigonal Bipyramid. *J. Chem. Soc. Dalt. Trans.* **2000**, No. 19, 3288–3303. https://doi.org/10.1039/b004878j.

(7) Alvarez, S.; Avnir, D.; Llunell, M.; Pinsky, M. Continuous Symmetry Maps and Shape Classification. The Case of Six-Coordinated Metal Compounds. *New J. Chem.* **2002**, *26* (8), 996–1009. https://doi.org/10.1039/b200641n.
